# Host biological sex directs immune control of the *Plasmodium* parasite liver stage in mice

**DOI:** 10.3389/fimmu.2026.1734587

**Published:** 2026-03-02

**Authors:** Caroline J. Duncombe, Alen S. Poehlman, Ethan N. Conrad, Nilasha Sen, Aaron Ayenew, Rebecca C. Blyn, GW McElfresh, Elizabeth K. K. Glennon, Kenneth Boey, Anya C. Kalata, Alexis Kaushansky, Nana Minkah, Melanie J. Shears, Sean C. Murphy

**Affiliations:** 1 Graduate Program in Pathobiology, Department of Global Health, University of Washington, Seattle, WA, United States; 2 Department of Laboratory Medicine and Pathology, University of Washington, Seattle, WA, United States; 3 Center for Emerging and Re-emerging Infectious Diseases (CERID), University of Washington, Seattle, WA, United States; 4 Center for Global Infectious Disease Research, Seattle Children’s Research Institute, Seattle, WA, United States; 5 Oregon National Primate Research Center, Oregon Health and Science University, Beaverton, OR, United States; 6 Department of Comparative Medicine, University of Washington, Seattle, WA, United States; 7 Department of Pediatrics, School of Medicine, University of Washington, Seattle, WA, United States; 8 Washington National Biomedical Research Center, University of Washington, Seattle, WA, United States; 9 Department of Microbiology, University of Washington, Seattle, WA, United States; 10 Department of Laboratories, Seattle Children’s Hospital, Seattle, WA, United States

**Keywords:** innate immunity, liver, malaria, Plasmodium, sex differences, testosterone, androgen, Kupffer cell

## Abstract

**Introduction:**

*Plasmodium* parasites, the causative agents of malaria, undergo a complex liver stage shaped by the spatial heterogeneity of the liver. As one of the most sexually dimorphic organs, the liver undergoes sex-specific immune responses. However, the roles of biological sex and androgens in regulating hepatic host–parasite interactions during *Plasmodium* infection remain poorly understood.

**Methods:**

Male and female BALB/cJ mice, including orchiectomized (ORX) and androgen-treated males, were infected with *Plasmodium yoelii* sporozoites. Hepatic parasite burden, immune cell composition, and gene expression were assessed using immunofluorescence microscopy, RT-PCR, and spatial transcriptomics. Comparisons across sex and androgen status were performed in infected and mock-infected conditions.

**Results:**

Androgens increased parasite survival by reducing elimination of parasite-infected hepatocytes. Females and ORX males exhibited enhanced immune activity during *P. yoelii* infection, resulting in fewer infected hepatocytes by 44 hours post-infection. At homeostasis, intact males had lower baseline densities of Kupffer cells, monocytes, and dendritic cells, a disparity that persisted and increased during infection. Although infection induced similar fold increases in Kupffer cell numbers across groups, intact males had reduced absolute numbers of Kupffer cells, diminished antigen presentation, and blunted Type I interferon responses compared to female and ORX male mice.

**Discussion:**

These findings indicate that biological sex- and androgen-mediated differences in baseline hepatic innate immune cell composition critically shape immune responses and parasite survival during *Plasmodium* liver stage infection. Together, our findings highlight the role of biological sex in shaping immune responses during *Plasmodium* parasite infection in the liver and underscore the importance of considering sex as a biological variable in malaria research.

## Introduction

*Plasmodium* spp., the unicellular eukaryotic parasites responsible for malaria, are transmitted to mammalian hosts through the bite of infected *Anopheles* mosquitoes (1). This transmission introduces *Plasmodium* sporozoites into the dermis, where the parasite is highly motile and travels through the host’s circulatory system to reach the liver via the portal vein (2). The sporozoite traverses across multiple hepatocytes, before establishing itself within a final hepatocyte. Upon entering a hepatocyte, the sporozoite forms a parasitophorous vacuole (PV), where it replicates rapidly in a process called schizogony while evading host defense mechanisms (3). *Plasmodium* parasites actively modify the host hepatocyte to access nutrients that support their growth and development (4–6). At the end of this transformation, the schizont form divides into merozoites, which are then released into the bloodstream, initiating the symptomatic blood stage of malaria (7).

The liver is a sexually dimorphic organ, characterized by sex-specific differential gene expression patterns and function (8), which in turn have implications for liver-tropic pathogen infections (9–11). During homeostasis, the male and female liver maintain differential expression of hundreds of genes in mice (12) and humans (13), including genes encoding xenobiotic and fatty acid metabolic enzymes, immune signaling pathways, and immune cell markers. Sex-specific patterns in function are most commonly characterized in metabolism (14, 15) and endocrine function, including sex hormone biosynthesis (16, 17).

Biological sex also modifies the spatial heterogeneity within the liver (18). The liver’s basic anatomical unit, the lobule, consists of layers of hepatocytes arranged around a central vein, forming a hexagonal structure (19). Hepatocytes, which make up about 80% of liver volume (20), radiate from this vein, with portal triads at each corner containing the portal vein, hepatic artery, and bile duct (21). To optimize metabolism, liver cells express different proteins along the axis from each portal node to the central vein, creating spatially defined metabolic zones known as zonation (22). Sex-specific gene expression also follows a zonal pattern in the lobule; for example, Goldfarb et al. found that 46% of zone-biased genes are sex-specific, particularly affecting pathways in lipid and xenobiotic metabolism (18).

Prior studies have mapped a spatially-resolved host response to liver stage infection with rodent *Plasmodium* parasites (23–25). These studies reveal information about the heterogeneity of host parasite interaction and immune activation during challenge that were not possible to observe using non-spatial techniques, such as flow cytometry, bulk or single cell transcriptomics analysis. For example, during *P. berghei* (*Pb*) infection, the host liver exhibits a spatially organized and sequential response, where interferon-driven immune gene activation emerges at later stages of parasite development (4, 26, 27). These immune patterns coincide with spatial differences in parasite growth dynamics, as replication rate and survival vary between pericentral (PC) and periportal (PP) hepatocytes (25). In addition, it has also been shown that immune cell infiltrates gather around infected hepatocytes following *Pb* infection (23, 26). Together, these insights have deepened our understanding of *Plasmodium* infection, hepatic zonation, and tissue-specific immune responses. However, despite the pronounced sexual dimorphism of the liver, these studies on *Plasmodium* liver stage heterogeneity have been conducted within a single sex. Thus, the influence of sex-specific factors and hormones on gene expression across hepatic zones, as well as the contribution of liver-resident immune cells during *Plasmodium* liver stage infection, remains unexplored.

In human adults, a recent pooled analysis of Controlled Human Malaria Infection (CHMI) studies found sex-based differences in the time to blood-stage positivity (longer in males), suggesting potential sex-specific effects during the *P. falciparum* (*Pf*) liver stage (28). Although the study did not explore underlying mechanisms, sex hormones—particularly androgens—have been implicated in shaping infection outcomes for other hepatotropic pathogens, notably, *Entamoeba histolytica* (9, 29), trematodes (30–33), and hepatitis viruses (10, 11, 34, 35), all of which tend to be more common or severe in males. In the case of amebiasis, the risk in males rises sharply after puberty and gradually declines with age (29). In mice, a recent study implicated a role for androgens in mediating vaccine-induced immune control of *P. yoelii* (*Py*) in the liver (36). Defining how androgens influence immune responses in the liver may clarify the biological basis of sex-specific CHMI outcomes and parallel observations in other hepatotropic infections.

Here, we examine the impact of biological sex on liver stage infection with the rodent parasite *Py*. We further characterize how androgens influence *Py* infection in male mice. To elucidate the effects of sex and sex hormones on the host response, we conducted spatial gene expression analysis of *Py*-infected mouse livers. We investigate genes, pathways, and immune cell dynamics influenced by biological sex (male vs female mice) and androgen levels (intact vs orchiectomized (ORX) male mice). We corroborate these findings through immunofluorescence microscopy. Altogether, this study provides an analysis of host-parasite interactions within the sexually dimorphic liver microenvironment.

## Methods

### Ethics statement

Animal studies were performed according to the regulations of the institutional animal care and use committee. Approval was obtained from the University of Washington Institutional Animal Care and Use Committee (IACUC) under protocol 4317-01. The University of Washington IACUC adheres to the NIH Office of Laboratory Animal Welfare standards (OLAW welfare assurance # D16-00292).

### Mice

Male and female BALB/cJ (4–6 wks old) were obtained from Jackson Laboratories (Barr Harbor, ME) and housed at the University of Washington in an IACUC-approved animal facility. All mice were used under an approved IACUC protocol (4317–01 to SCM). Primary euthanasia was conducted with CO_2_ inhalation at 30-70% of chamber volume per minute, followed by a secondary dissection method.

### *Plasmodium* sporozoites

Female *Anopheles stephensi* mosquitoes infected with wild-type *Py* (strain 17XNL) were reared at Seattle Children’s Research Institute (Seattle, WA) or UW Medicine (Seattle, WA). Fresh sporozoites (spz) were obtained by salivary gland dissection 14–18 days post-infection. All spz were diluted in Schneider’s insect media for administration (Gibco, Thermo Fisher). Figure legends specify the dose for each experiment. For *Py* wild-type (WT) spz administrations, 1x10^5^ freshly dissected *Py-*WT spz were injected intravenously (IV, retro-orbital) unless stated otherwise. Each independent replicate involved a distinct mosquito cage and independently administered parasite infection.

### Tissue processing for microscopy

Following infection with *Py* or mock injection with loading media (Schneider’s), livers from mice were harvested at 12, 24, or 44 hours post infection (hpi) and left lateral lobe was fixed in 10% neutral buffered formalin solution (Millipore Sigma) for 24 hours then transferred to 70% ethanol. Tissues were then paraffin embedded, cut into 5 µm sections, and mounted on positively charged glass slides. Mounted liver slices were then used for digital spatial profiling or immunofluorescence staining.

### Immunofluorescence staining

Slide-mounted liver slices were washed twice in xylene (Fisher Scientific) for 3 minutes followed by washes in 100% (twice), 95%, 70% and 50% ethanol for 3 minutes each. Slides were then washed with DI water and heated to 90°C for 30 minutes in 1% citrate-based antigen unmasking solution (Vector Laboratories) using a Decloaking Chamber (Biocare Medical). After primary and secondary antibody staining, autofluorescence was quenched using Vector TrueView (Vector Laboratories), and Fluoromount G (Southern Biotech) was applied for mounting.

For Kupffer cell localization experiments and quantification of spatial localization and parasite size at 24hpi, slides were washed with Tris-buffered saline (TBS) + 1% Triton X-100 (Sigma Aldrich), blocked for 2 h in 1.5% Bovine Serum Albumin (BSA; GoldBio) + 15% goat serum (Sigma Aldrich), and incubated with PyHSP70 antibodies overnight at 4 °C. After washing, secondary antibodies and DAPI were applied for 1 h at room temperature. Because CLEC4F antibodies are mouse-derived, slides were re-blocked and incubated with conjugated CLEC4F-AF647 antibodies overnight. Antibody concentrations: *Py*Hsp70 (gift from Kaushansky Lab) 1:500, CLEC4F-AF647 (BioLegend 156804) 1:100, anti-mouse AlexaFluor-488 (Thermo Fisher Scientific A-11001) 1:1000.

For quantification of spatial localization and parasite size at 44 hpi, blocking was performed in PBS containing 0.3% Triton X-100 (Sigma Aldrich), 1% BSA (Fisher Scientific), and 10% natural goat serum (Jackson ImmunoResearch) for 1 h. Slides were incubated for 1 h at room temperature with primary antibodies plus Hoechst (1:1000; Invitrogen) and DAPI (1:400; Invitrogen), followed by PBS washes and secondary antibody incubation (1 h, room temperature). Antibody concentrations: PfHsp70 (StressMarq SPC-186; cross-reacts with *Py*) 1:500, anti-ASS1-AF488 (Abcam ab208412) 1:250, anti-GS (Abcam ab64613) 1:500; secondary antibodies anti-rabbit AlexaFluor-594 (Invitrogen A32740) and anti-mouse AlexaFluor-647 (Invitrogen A32728), both at 1:500.

### Imaging and quantification

**Figure 1:**
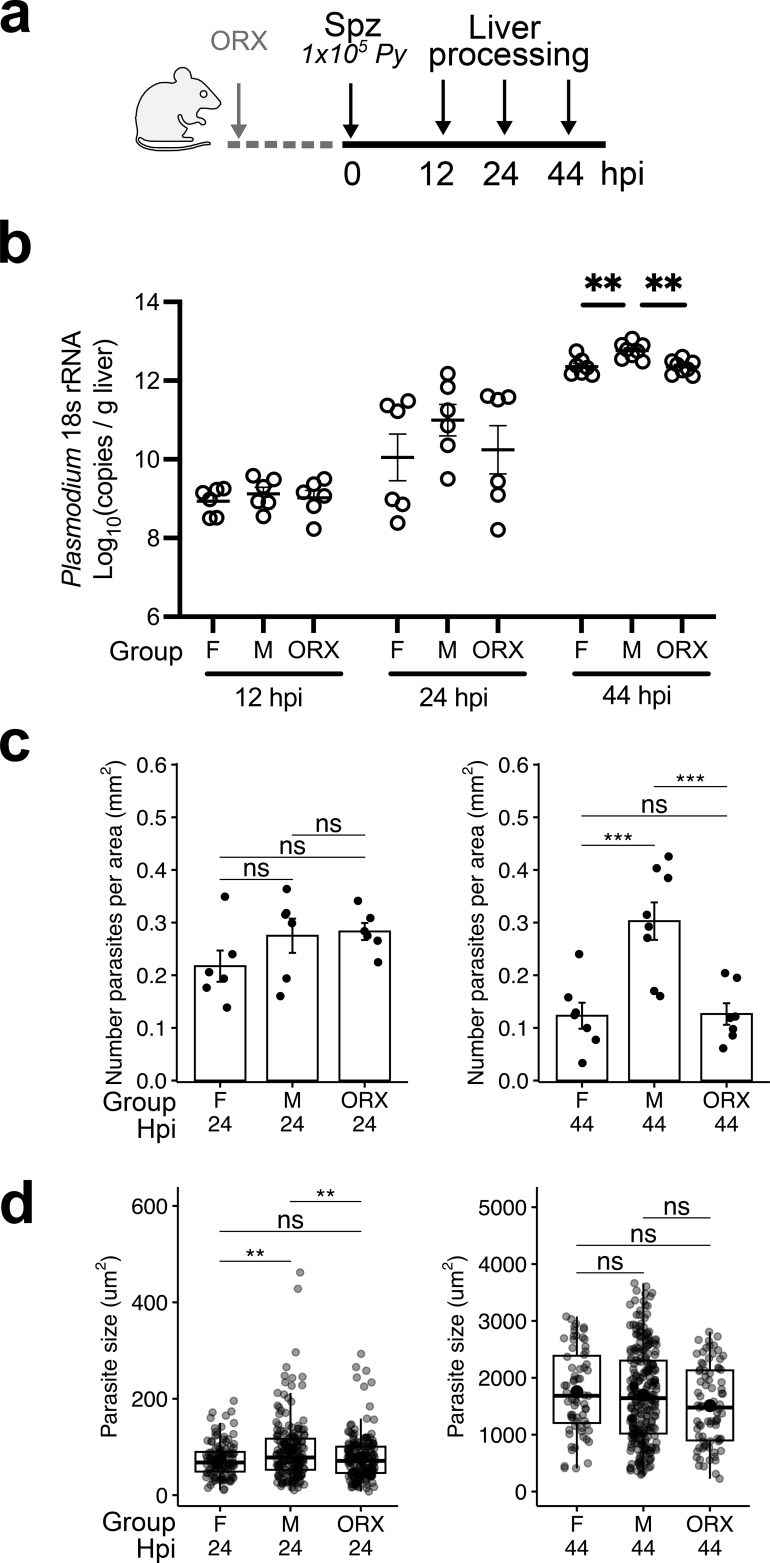
Sex bias in survival of *Plasmodium yoelii* infection in BALB/cJ mice. **(a)** Schematic of male (M), female (F), and orchiectomized male (ORX) BALB/cJ mice infected with 1x10^5^*Py*-WT spz intravenously (IV) and harvested at 12, 24, and 44 hours post infection (hpi). **(b)** Livers were excised for absolute pan-*Plasmodium* 18S rRNA quantification at 12 hpi, 24 hpi and 44 hpi. **(c)** Density of liver stage parasites per area of liver tissue quantified by microscopic counting at 24 hpi (left) and 44 hpi (right). **(d)** The size of liver stage parasites was quantified based on area of HSP70 stain with fluorescence microscopy at 24 hpi (left) and 44 hpi (right). Data are shown from two independent experiments (n= 6–8 mice). Data were analyzed by one-way ANOVA with Tukey multiple comparison. Error bar represents mean ± s.e.m; box plot depicts median with interquartile range. ***p<0.001, **p<0.01, ns p≥0.05.

For Kupffer cell localization experiments, images (40X) were acquired using a high-resolution widefield microscope (Nikon) focused on infected hepatocytes and uninfected regions. For cell quantification within regions of interest (ROIs), 4x4 image panels were taken with a 60-pixel overlap and stitched together using the DAPI channel. Images were stitched and deconvolved using the NIS-Elements software (Nikon) and were visualized using ImageJ/FIJI software (37, 38). ImageJ was used to quantify fluorescence intensity within defined ROIs of 200μm radii. Distances from parasitized cells to Kupffer cells (KCs) were measured between nucleus centers. Only KCs with a visible, DAPI-stained nucleus and a clear surrounding CLEC4F signal were included in counts. Whole lobe images were also captured on the 24hpi samples in **Figures 1c, d**. In this case, whole lobe images were taken with a high-resolution widefield microscope (Nikon) at 10X with 60-pixel overlap and stitched together using the DAPI channel. Only parasites with visible, DAPI-stained nuclei were included in counts and measurements.

For quantification of spatial localization and parasite size at 44 hpi, images (20X) were acquired and stitched using an Automated Microscope (Keyence). For liver zonation, periportal, interzonal, and pericentral regions were identified by ASS1 and GS staining. Periportal regions were classified as ASS1^hi^GS^lo^, interzonal regions were classified as ASS1^int^GS^lo^, and pericentral regions were classified as within 3 nuclei of ASS1^lo^GS^hi^. Images were visualized for analysis using ImageJ software. ImageJ was used to count the number of parasites in each liver region and define ROIs around parasites to measure parasite area. Only parasites with visible, DAPI-stained nuclei were included in counts and measurements.

### Liver burden quantification by *Plasmodium* 18S rRNA RT-PCR

To quantify liver burden by reverse transcription polymerase chain reaction (RT-PCR), mice were sacrificed, half of the liver was excised and pulverized by bead beating in NucliSENS lysis buffer (bioMérieux, Durham, NC, USA), and nucleic acid was extracted on an EasyMAG instrument (bioMérieux). RNA was subjected to RT-PCR using the SensiFAST™ Probe Lo-ROX Kit (Bioline, London, UK) using a predesigned HEX-labelled mouse GAPDH RT-PCR assay (IDT Inc, Coralville, IA) multiplexed with a Pan-*Plasmodium* 18S rRNA RT-PCR assay as described (39). RT-PCR conditions of 45 °C for 10 min, 95 °C for 2 min and 45 cycles of 95 °C for 5 s, 50 °C for 35 s were performed on a QuantStudio 5 real-time PCR machine (Thermo Fisher Scientific). Copy numbers per reaction were determined using custom lot of quantified Armored RNA encoding full-length *Plasmodium* 18S rRNA (Asuragen, Austin, TX).

### Acyline and testosterone administration

Acyline (lot # RDZ001) was provided by John K. Amory, MD, MPH at the University of Washington. Lyophilized acyline vials were stored at -20 °C until resuspension. At the time of use, acyline was resuspend to a concentration of 2 mg/mL with sterile Molecular Biology Grade Water (Cytiva). Resuspended vials were stored for a maximum of 1 week at 4 °C. Mice were subcutaneously injected with 300µg of acyline on 5 and 4 days prior to infection to give time for the hormone environment to equilibrate (36). For testosterone treatment, testosterone propionate (Sigma Aldrich) was dissolved in sesame oil (Sigma Aldrich) at a concentration of 20 µg/µL. Mice were weighed to determine average weight per group, and then subcutaneously injected with 100 µg testosterone/g body weight at 2 timepoints (3 days and 1 day prior to infections).

### Testosterone quantification in serum

Serum was collected via chin bleed day of harvest and stored in -20 °C until testing. To measure testosterone levels in serum, Testosterone ELISA kits (Crystal Chem) were used per manufacturer guidelines. Singlet absorbance values of each sample were measured with CLARIOstar Plus Microplate Reader (BMG Labtech) and analyzed using MARS data analysis interface. A calibration curve was generated using manufacturer provided standards (0,0.1, 0.4, 1.5, 6.0, and 25 ng/mL) and a four parametric logistic (4-PL) curve fit was used to calculate sample values.

### Orchiectomy

Male mice were orchiectomized at least 30 days prior to treatment. Male mice were anesthetized by isoflurane inhalation (Isoflurane, USP, Dechra Veterinary Products), with doses of 4-5% for induction and, 2-3% for maintenance. A 5 mg/kg dose of meloxicam (Pivetal^®^ Alloxate™, Aspen Veterinary Resources^®^, LTD) was administered subcutaneously (SC) to each mouse for systemic analgesia. Lubricant ophthalmic ointment (Pivetal^®^ Artificial Tears, Aspen Veterinary Resources^®^, LTD) was applied to both eyes to prevent corneal desiccation. Thermal support was provided using a warm water circulating heating pad. The incision site was surgically prepped with three alternating scrubs of 10% povidone-iodine and 70% ethanol. A small, midline, scrotal incision was made with tissue scissors. Each testicle was identified singly, and the associated spermatic cord was clamped with hemostats for at least 10 seconds, and the testicle removed. The incision was closed using surgical tissue adhesive (3M™ Vetbond Tissue Adhesive 1469, 3M). Sham ORX surgeries were performed using the same surgical protocol without surgical removal of the testicles.

### Digital spatial profiling

Spatial transcriptomics was conducted using the GeoMx Digital Spatial Profiler (Nanostring) with a previously described protocol (40). Tissues were cut onto positively charged slides at a 5μm thickness, dried overnight, and baked at 65 °C for 1 hour. All subsequent steps were performed in an RNAse-free environment with diethyl pyrocarbonate (DEPC) water. Slides were de-paraffinized with xylene, rehydrated using graded alcohols, and held in 1X PBS for 1 minute. Tissues were incubated in Tris/EDTA pH 9.0 antigen retrieval solution (Invitrogen #00-4956-58) pre-heated to 100 °C for 15 minutes, washed in 1X PBS for 5 minutes, and transferred to 37 °C 1.0 µg/ml proteinase K (Ambion #AM2546) for 15 minutes. Slides were washed in 1X PBS for 5 minutes, then post-fixed with a 5-minute incubation in 10% neutral-buffered formalin (Epredia #5701) followed by two 5-minute incubations in Tris-glycine fixation stop buffer (Sigma #77-86-1, #G7126) and one 5-minute wash in 1X PBS. Slides were hybridized overnight at 37 °C with the Mouse Whole Transcriptome Atlas probe set (Nanostring #121400313) diluted in Buffer R (Nanostring #121300313) and DEPC water. Unbound probes were removed using two 25-minute incubations in stringency wash buffer comprised of 50% deionized formamide (Sigma #4650) and 50% 4X Saline Sodium Citrate (SSC) buffer (Sigma #S6639). Slides were washed twice with 2X SSC buffer for 2 minutes, then incubated in Buffer W (Nanostring #121300313) for 30 minutes. Slides were stained overnight in morphology marker *Pf*HSP70 antibody (Rabbit polyclonal, 1:42 dilution, StressMarq #SPC-186D), washed twice with 2X SSC for 5 minutes, and detected with a 1 hour incubation in secondary antibody conjugated to AlexaFluor 647 (Goat polyclonal, 1:200 dilution, Jackson ImmunoResearch #111-605-144). This was followed by two 5 minute 2X SSC washes and a 1-hour incubation of labeled antibodies ASS1-AlexaFluor 488 (Rabbit clone EPR12398, 1:250 dilution, Abcam #ab208412) and GS-AlexaFluor 594 (Mouse clone 3B6, 1:60 dilution, Abcam #ab64613) cocktailed with nuclear counterstain Syto83 (2 uM, 1:10, Invitrogen #S11364). Slides were held overnight in 2X SSC buffer, then re-stained for 10 minutes with Syto83 immediately prior to loading the instrument.

Following whole-slide fluorescent imaging, circular ROIs were placed on tissue areas. Syto83 immunofluorescence was utilized for autofocus of GeoMx imaging. Immunofluorescence for ASS1 was used to identify periportal (PP), pericentral (PC), and interzonal (IZ) regions and *Pf*HSP70, which cross reacts with *Py*, was used to identify the location of parasites. ROIs were generated using the circle tool and measured to a diameter of 200 µm, 100 µm, 75 µm, and 50 µm. Each ROI was selectively exposed to UV light, cleaving the barcode sequences and collecting them in a unique well in a 96-well collection plate (three plates in total). Libraries were prepared with Nanostring SeqCode construction kits according to manufacturer instructions, sequenced using NovaSeq 6000 SP flowcell, and processed using the GeoMX NGS Pipeline.

### Statistics and reproducibility

#### Quality control

In the GeoMx experiment, 278 segments were collected from 18 mice, and 5 of these segments failed Quality Control (QC) analysis and were removed from the dataset. One segment was removed for sequence stitching <80% and aligned sequence reads <80%. Two additional segments were removed for aligned sequence reads <80% alone, and two segments were removed for sequence saturation <50%. A secondary blinded reviewer conducted a follow-up quality control (QC) evaluation of the liver zones after initial collection. ROIs with identified discrepancies in liver zones of ROI in accordance between initial and secondary reviewers were excluded from subsequent analyses (14 ROIs removed). Additionally, we excluded 60 ROIs with diameters that deviated from the specified 200 µm diameter threshold and were collected for exploratory purposes. Final analysis included 199 ROIs (**Supplementary Table 1**).

Of the 19963 non-housekeeping gene targets contained in the GeoMx Mouse Whole Transcriptome Atlas, all were included in the downstream analyses following QC analysis.

#### Normalization and batch correction

The dataset was processed using the Seurat package. Gene expression data were normalized using Seurat’s NormalizeData function (scale.factor = 10,000), which performs a library size normalization and log-transforms the data (41). Variable features were identified with the FindVariableFeatures function, selecting the top features for downstream analysis. The selected features were then scaled using the ScaleData function to standardize expression values across cells.

To correct for batch effects, raw count data were extracted from the Seurat object and converted into a matrix format compatible with the Surrogate Variable Analysis (SVA) package (42). Batch variables were defined based on the experimental block, and covariates (sex/hormone status and infection status) were incorporated into a covariate matrix. Batch correction was performed using the ComBat_seq function from the SVA package, and the adjusted count matrix was then used for subsequent analyses.

#### Clustering analysis

Clustering was performed the Seurat dataset integration pipeline with Louvain clustering. Using the Seurat functions: NormalizeData, FindVariableFeatures (nfeatures = 2000), FindIntegrationAnchors (dim = 1:15), IntegrateData (dims = 1:30, k.weight = 50), ScaleData, RunPCA (npcs = 30), RunUMAP (dims = 1:8), FindNeighbors (dims = 1:8), FindClusters (resolution = 1.3). Unless otherwise specified, all other values were left at default. Elbow plot of standard deviation explained by principal components was used to determine UMAP and FindNeighbors dim values (**Supplementary Figure S7d**).

### Differential expression

Differential expression of gene targets was determined with DESeq2 package (43). The regression model implemented was as follows:

(1) $$gene=\beta_{1}Group+\beta_{2}Type+\beta_{3}Liver\;zone+\beta_{4}Group\times \;Liver\;zone+\;\beta_{5}Group\times Type$$

Where Group is a designation for sex/hormone status of mouse (eg. male (M), female (F), and orchiectomized male (ORX)); Type is a designation for the ROI’s infection type (e.g. mock, bystander, and parasite); and Liver zone is a designation for liver zone (e.g. periportal (PP), interzonal (IZ), pericentral (PC)).

In order to quantify the joint effect of Group and Type using GSEA, we concatenated the Group and Type variables leading to the simplified version of the previous regression formula, omitting the bystander effects to focus the effects between mock and parasite stratified by group and controlling for liver zone:

(2) $$gene=\beta_{1}Group\_Type+\beta_{2}Liver\;zone\;\;$$

Where Group_Type is a combined designation for sex/hormone status and infection type (e.g. F mock, F parasite, M mock, M parasite, ORX mock, ORX parasite); and Liver zone is a designation for liver zone (e.g. periportal (PP), interzonal (IZ), pericentral (PC)).

In order to provide an exhaustive set of differential expressed genes between all comparison, a fully stratified set of comparison for each independent variable (Group, Type, Zone) individually is included in the supplements (**Supplementary Figures S9–S12**).

Differentially expressed genes (DEGs) were defined as genes with an adjusted p-values (false-discovery rate) < 0.05 and |Log2 Fold Change| > 1.0.

### Pathway analysis

Hypergeometic enrichment tests against Broad MSigDBHallmark (H) and KEGG (K) pathway gene sets (44, 45) were performed to determine pathways with significant overrepresentation. Gene set enrichment analysis (GSEA) was performed again H and KEGG gene sets with contrast fold changes using fast GSEA (46).

### Cell-type deconvolution

We used BayesPrism’s (47) (via InstaPrism (48)) cell-type deconvolution algorithm to determine the cell-type proportions in our spatial transcriptomics data. We determined cell-type proportions by deconvoluting our data with single-nucleus data from Hildebrandt et al. (23), which was conducted on C57BL/6 mice during *Pb* infection and included 14 cell types. The gene expression profiles for deconvolution were computed via genes averaged across each cell types within the snRNASeq dataset. To determine statistical significance of difference in cell type proportions, we compared groups with a Wilcoxon test with Benjamini–Hochberg adjusted p-values.

### Statistics

Details of the statistical tests applied to datasets shown in figures can be found in the above methods and in corresponding figure legends. All data points and n values reflect biological replicates. Data analyzed included outliers unless these could be explained by technical error. GeoMx data collection and gene expression analysis were not performed blind to the conditions of the experiments. Fluorescent microscopy with CLEC4F antibody and liver zonation were counted blinded to experimental conditions. Fluorescent microscopy and *Plasmodium* 18S rRNA RT-PCR data were assumed to be normal thus parametric tests were employed. Where relevant, statistical tests were two-sided. Individual p-values are provided in the figures. Statistical analyses were performed in R (version 4.3.2) or GraphPad Prism 9.1.2 Software. Plots used in this manuscript were generated with GraphPad Prism and the following R packages: ggplot2 (49), ggpubr (50), ComplexHeatmap (51), and UpSet (52).

## Results

### Biological sex and androgens impact *Plasmodium yoelii* liver stage survival

To test if biological sex modify susceptibility to infection or subsequent liver stage development, we infected adult male and female BALB/cJ mice with 1 x10^5^*Py* spz intravenously (IV). Livers were isolated at 12, 24, and 44 hours post infection (hpi) for quantification of *Plasmodium* parasite RNA by RT-PCR (**Figure 1a**). At 12 and 24 hpi, no significant sex difference was observed. However, at 44 hpi, males had an approximately 3-fold higher liver burden compared to female age-matched mice (**Figure 1b**), indicating a sex difference in parasite growth or survival.

Given the established role of androgens in modulating the pathogenesis of hepatotropic infections (9) and recent evidence of androgen-mediated effects on immune responses to malaria liver stage vaccination (36, 53), we hypothesized that androgens play a role in regulating increased liver stage parasite burden during *Plasmodium* infection. To test this, we orchiectomized (ORX) adult male mice at least 14 days prior to spz injection and quantified liver burden as described above (**Figure 1a**). ORX is a common method for removing testicular hormones, and is frequently applied to reduce androgen levels in murine systems (54). ORX males exhibited reduced liver burden at 44 hpi, but not at 12 or 24 hpi (**Figure 1b**) when compared to intact male mice. The apparent bimodality observed at 24 hpi reflects differences between two independent experimental batches rather than biological heterogeneity. To further assess the role of androgens in regulating parasite survival, we employed an acute and reversible model to suppress testosterone production in male mice. Acyline is a gonadotropin releasing hormone (GnRH) antagonist that suppresses downstream production of testosterone in a dose-dependent manner and has previously been applied in mice (55), and humans (56, 57). We depleted testosterone with acyline prior to *Py* infection and measured liver burden at 44 hpi (**Supplementary Figures S1a, b**). We found reducing testosterone was sufficient to decrease liver burden outcomes in male mice. Testosterone repletion before infection restored *Plasmodium* 18S rRNA levels relative to acyline-only (testosterone-depleted) group (**Supplementary Figure S1c**), confirming its association with liver burden. Together, these findings lead us to conclude that androgens significantly influence parasite burden during the liver stage.

At least two factors could influence the quantity of *Plasmodium* rRNA at 44 hours: 1) the number of infected hepatocytes or 2) the rate of parasite replication within hepatocytes. To evaluate if sex influenced these factors, the number of infected hepatocytes was counted and parasite size was measured by fluorescence microscopy. There were more parasites in males compared to females and ORX males at 44 hpi, but not at 24 hpi (**Figure 1c**). Parasite size was not significantly different between groups at 44 hpi (**Figure 1d**), even when stratifying by liver zone (**Supplementary Figure S2**). Interestingly, parasite size was modestly, but significantly greater in intact males at the 24-hour timepoint, indicating that initial rate or initiation of replication might differ. Taken together, these data suggest that the higher liver burden at 44 hpi in males compared to females and ORX males reflects parasite survival rather than initial invasion or parasite replication rate.

### Spatial transcriptomics captures sex-specific responses induced by *Plasmodium* parasite infection

**Figure 2:**
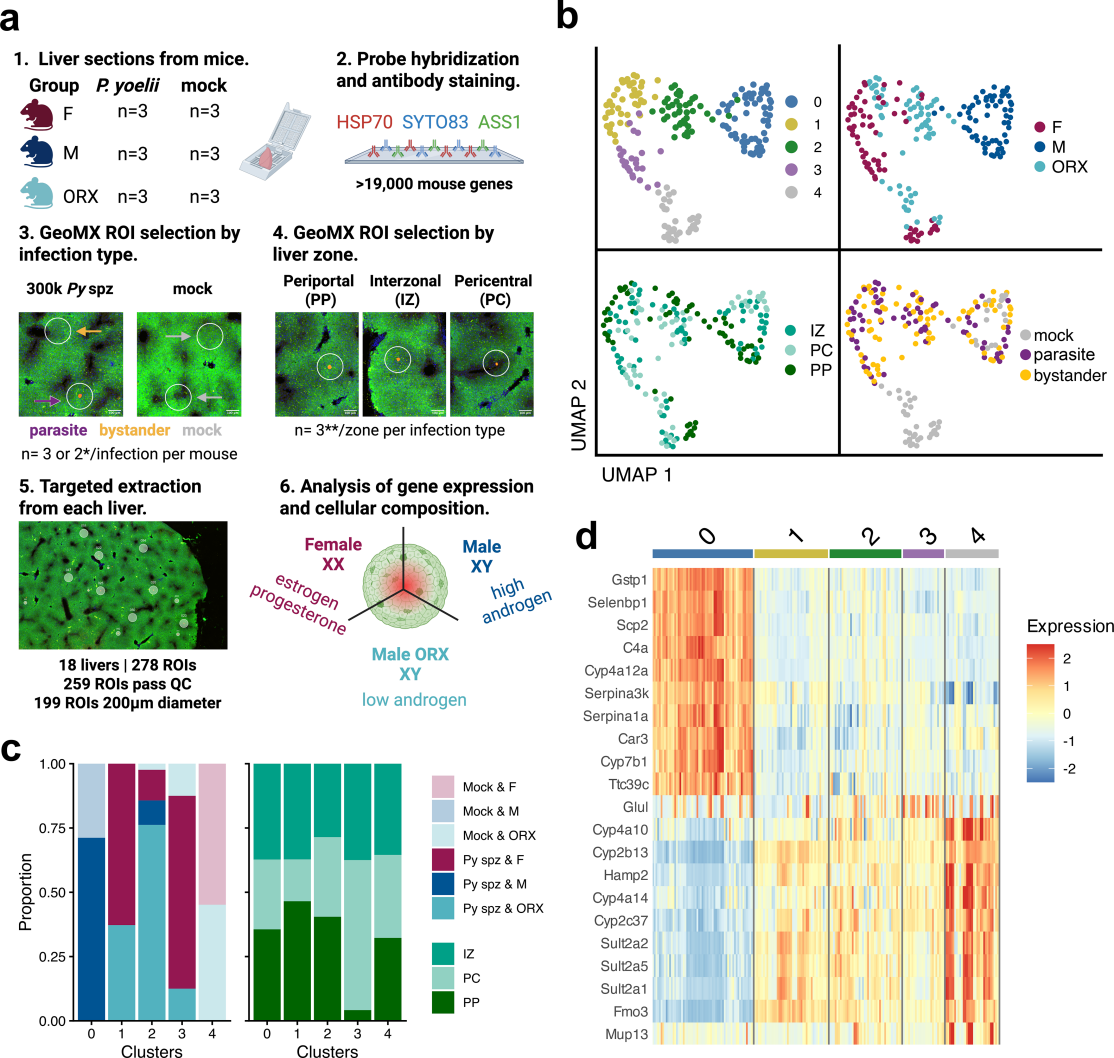
Digital spatial profiling facilitates evaluation of transcriptomes in *Plasmodium yoelii* infected tissues. **(a)** The workflow to evaluate fixed liver samples from BALB/cJ mice infected with *Py* sporozoites. 1) Biopsies from 18 mice were fixed and assembled to evaluate mice challenged with 3x10^5^*Py* spz IV (3 per group) or mock infected (3 per group) in three groups, adult female (F), male (M), and orchiectomized male (ORX) mice. 2) Liver sections were stained with anti-ASS1, anti-SYTO83, and anti-HSP70 to visualize the liver zone and the presence of *Plasmodium* parasites and then hybridized with a >19,000 RNA probe panel targeting mouse genes for GeoMX assay. 3 - 4) Region of interests (ROIs) were selected based on the infection status of the mouse and the zonal location of the parasite. To ensure representation from each zone, at least 3 ROIs were selected per infection type (bystander, parasite, mock) and per liver zone (PC, IZ, PP). 5) Targeted extraction of whole transcriptomes within each region enables evaluation of different regions from mouse groups. 6) Computationally dissect the key differences underlying response to infection by biological sex and hormone status. *Py spz samples had 3 ROIs per infection type and mock samples had 2 each. **Up to 3 zones were captured if enough parasites were present at time of collection. Created with BioRender.com. **(b)** The normalized and batch-corrected data were embedded in Uniform manifold approximation and projection (UMAP) space and colored by clusters identified through unsupervised clustering and phenotypes. **(c)** Proportion of ROIs of annotated conditions (infection status, sex and hormone status, and zonal location) within clusters identified in **(b, d).** Heat map of top 10 most significant genes (with a |Log_2_ Fold Change| > 1) associated with each cluster identified in b.

To interrogate the impact of sex and sex hormones on the host response to *Plasmodium* infection, we performed GeoMx spatial genome-wide transcriptomic profiling on *Py-*infected BALB/cJ mouse liver tissue. Adult intact male, female, and ORX male mice were infected IV with 3 x10^5^*Py* spz or mock injected. The comparison with mock-infected mice enabled us to evaluate the baseline contributions of biological sex in this model. At 44 hpi, liver sections from the left lateral lobe were collected and prepared for digital spatial profiling. Regions of interest (ROIs) were selected based on staining with three markers: one for *Plasmodium* parasites (HSP70, a cytosolic marker), one for liver zonation (argininosuccinate synthetase 1, ASS1, which marks the periportal region of the liver), and one nuclear stain (Syto83). ROIs were selected in tissue from infected and mock infected mice. In infected mice, ROIs were selected to capture both local and distal immune responses—one encircling the infected hepatocyte and its immediate neighboring cells and another bystander region positioned away from any detectable parasites (**Figure 2a**). As zonal location (Periportal (PP), Interzonal (IZ), and Pericentral (PC)) is known to influence the host response to infection (23, 25), we selected around 3 ROIs per mouse per zone per infection type (**Supplementary Table S1**) and confirmed expected zonal gene expression phenotypes (**Supplementary Figure S3**).

First, we performed unsupervised clustering to identify overall trends in the data. We identified 5 clusters (0–4) (**Figure 2b**). Many of these clusters represented the hormone status of the mouse, with intact males (Cluster 0) differentiating from females (Clusters 1, 3, 4) and ORX males (Clusters 1, 2, 4) regardless of infection status (**Figure 2c**). Clusters associated with females and ORX males primarily differentiated on infection status (Cluster 4 – uninfected; Cluster 1, 2, and 3 – infected). In contrast, infected and uninfected intact males primarily clustered together (Cluster 0). We found genes enriched in female and ORX clusters were connected to the cytochrome P450 superfamily, fatty acid metabolism, and hormone biosynthesis (**Figure 2d**). Cluster 3 captured liver zone-specific expression with enrichment more in the PC and IZ regions (**Figure 2c**). With these clusters defined, we next conducted a supervised analysis to further investigate the impact of biological sex, liver zone, and infection type on gene expression outcomes.

### Sex-bias in gene expression in the liver at steady state

**Figure 3:**
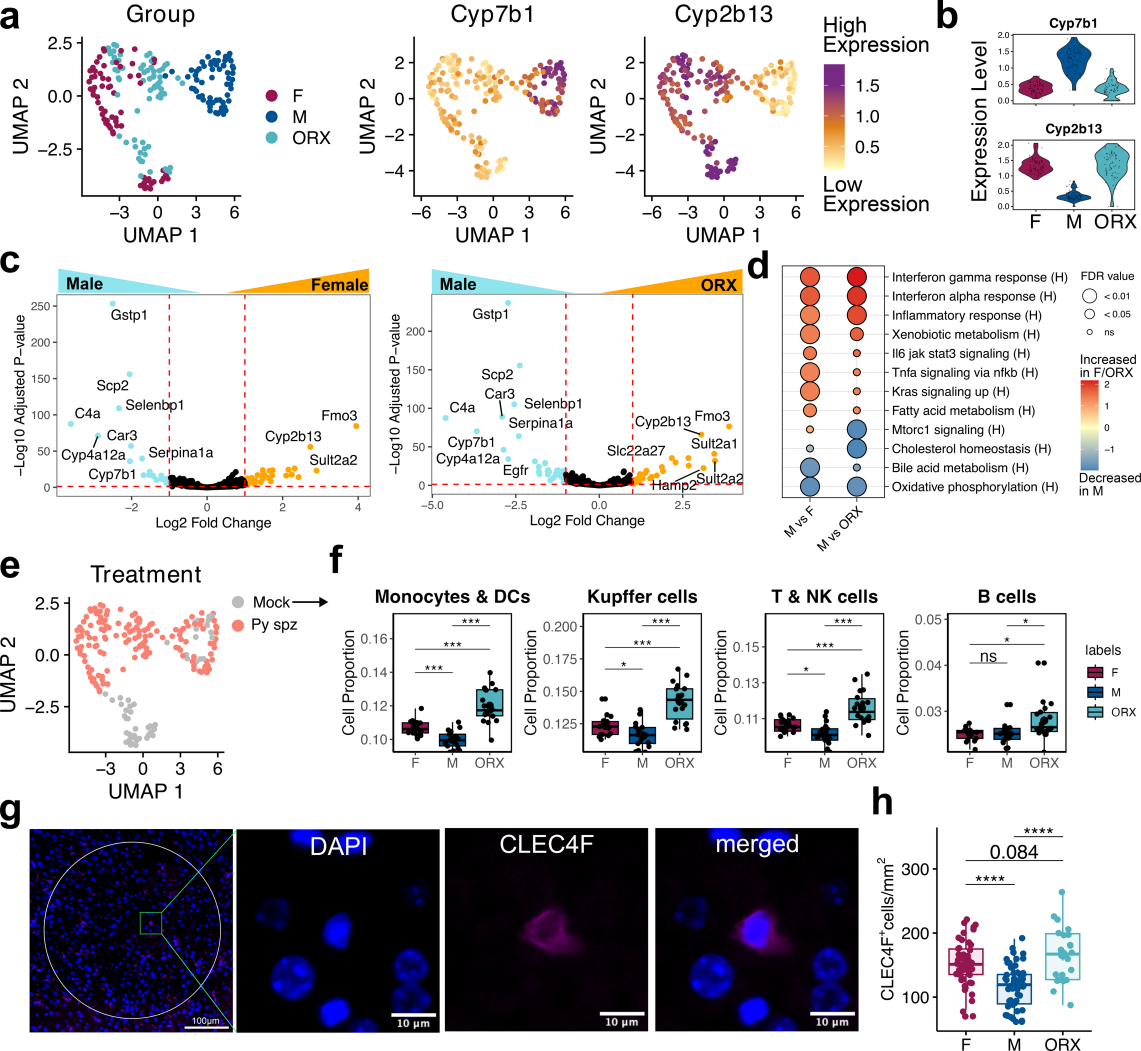
Global sex bias in immune cell density and gene expression. **(a)** UMAP of ROIs, colored by biological sex and hormone status (left), and by expression of the male-biased gene *Cyp7b1* and androgen-naïve biased-gene *Cyp2b13*. **(b)** Violin plots of expression levels (counts per 10,000 reads) of selected genes. **(c)** Volcano plot showing differentially expressed genes across all females (F) and males (M) (left) and orchiectomized males (ORX) and intact males (right), based on β_1_ in Equation 1. The y-axis shows the log_10_ adjusted p-value. The x-axis shows the log_2_ fold change. Genes significantly increased or decreased in ROIs are plotted in orange or blue, respectively (adjusted p-value < 0.05 and Log_2_ Fold Change +/- 1). **(d)** Gene set enrichment analysis (GSEA) between F and M, and ORX and M. **(e)** UMAP of ROIs, colored by infection status. **(f)** Mock infected mice-restricted cell type deconvolution of ROIs, separated by biological sex and hormone status. Statistical analysis performed via unpaired Wilcoxon tests with BH-correction. **(g)** Representative image of a Kupffer cell (DAPI^+^CLEC4F^+^) in mock-infected liver tissue collected at 44 hpi. Ring captures a 200μm radius randomly selected within a mock-infected tissue. **(h)** Number of CLEC4F^+^ cells normalized by area in mock-infected liver tissue (two independent replicates, n = 5 – 8 mice; up to 10 circles per mouse; one-way ANOVA with Tukey multiple comparison). Box plots depict median with interquartile range. ****p <0.0001, ***p <0.001, **p<0.01, *p<0.05, ns p>0.05.

To evaluate how sex and sex hormones impact the liver independent of liver zonation and infection type, we overlaid phenotypic information of all ROIs collected. Globally, male mice differentiate from female and ORX male mice with high expression of *Cyp7b1*, a cytochrome p450 family gene that is known to be regulated by biological sex in the murine liver (58). Female and ORX male mice also differentiate from intact male mice with higher expression of *Cyp2b13*, a gene connected to lipid and fatty acid metabolism (59) (**Figures 3a, b**). We were able to capture known differences in gene expression by biological sex and hormone status (18, 58, 60), and identified that clustering is principally determined by androgen presence, rather than biological sex.

To identify differentially expressed genes at steady state, we performed pairwise comparisons between all intact males and females, and intact males and ORX males, while accounting for covariates of infection and liver zone (Equation 1, β_1_). We found most genes differentially expressed between females and intact males were also differentially expressed between ORX and intact males (**Figure 3c**; **Supplementary Figure S4**). Upregulated genes in females and ORX males were enriched for genes associated with fatty acid metabolism (e.g. *Acot3*, *Hao2*) and xenobiotic metabolism (e.g. *Fmo3*). Upregulated genes in males were linked to bile acid metabolism (e.g. *Cyp7b1*, *Scp2*) and complement/coagulation cascades (e.g. *C4a, C9*) (**Figure 3d**; **Supplementary Figure S4**), corroborating previously identified biases (61).

We next explored if biological sex and hormone status modified gene expression patterns by liver zone. Previous studies have identified sex-bias gene expression patterns by liver zones (18, 62). We identified three significantly differentially expressed genes, using an interaction term between sex and liver zone on all mice, that were modified by biological sex and hormone status and downregulated between PC and PP region: *Cyp2c29, Csad*, and *Cyp2a4* (**Supplementary Figure S5**). While previous single-cell studies have shown that biological sex can influence liver zone-specific gene expression, the fact that GeoMX DSP ROIs represent multiple types of cells within the liver may have diluted this signal.

### Sex-based differences in liver cellular composition at baseline

To estimate cell type proportions for each ROI in the spatial gene expression data we leveraged a single-nucleus RNAseq data set from livers of C57BL/6 mice infected with *Pb* published by Hildebrandt et al. (23). This dataset defined liver cell populations through cluster-based gene expression profiles, which we used for deconvolution (47, 48). We focused on mock-infected groups to define cellular composition by sex in the liver at baseline (**Figure 3e**). Comparing the proportions of 14 annotated cell types, we found that within our selected ROIs that hepatocytes comprised 20 – 40% and remaining cells type comprised 60 – 80% of the RNA measured in the tissue (**Supplementary Figure S6**). We note this was different than Hildebrandt et al., likely due to differences between *in situ*- and droplet-based sequencing platforms, divergent mouse strains, and snRNAseq method versus targeted probe-based sequencing. Four immune cell categories were captured with this method: KCs, monocytes and dendritic cells (DCs), T and NK cells, and B cells. Analysis of cell proportions revealed that ORX males had the highest levels of KCs, monocytes, DCs, and T and NK cells, followed by females, with intact males exhibiting the lowest levels (**Figure 3f**). Thus, this analysis recapitulates similar sex-specific differences in baseline immune cell proportions in tissue as previously identified in rodent models (63, 64).

To confirm the finding that females and ORX males have a higher density of KCs compared to males in mock-infected tissues, we used fluorescent microscopy and quantified KCs with a CLEC4F marker (**Figure 3g**). We found that females and ORX males have higher baseline levels of CLEC4F^+^ cells compared to the males (**Figure 3h**). Overall, this suggests that differences in baseline gene expression and immune cell composition between intact male and female mice are strongly influenced by androgen status, establishing a distinct metabolic and immune homeostatic state.

### Biological sex impacts inflammatory response to *P. yoelii* infection

**Figure 4:**
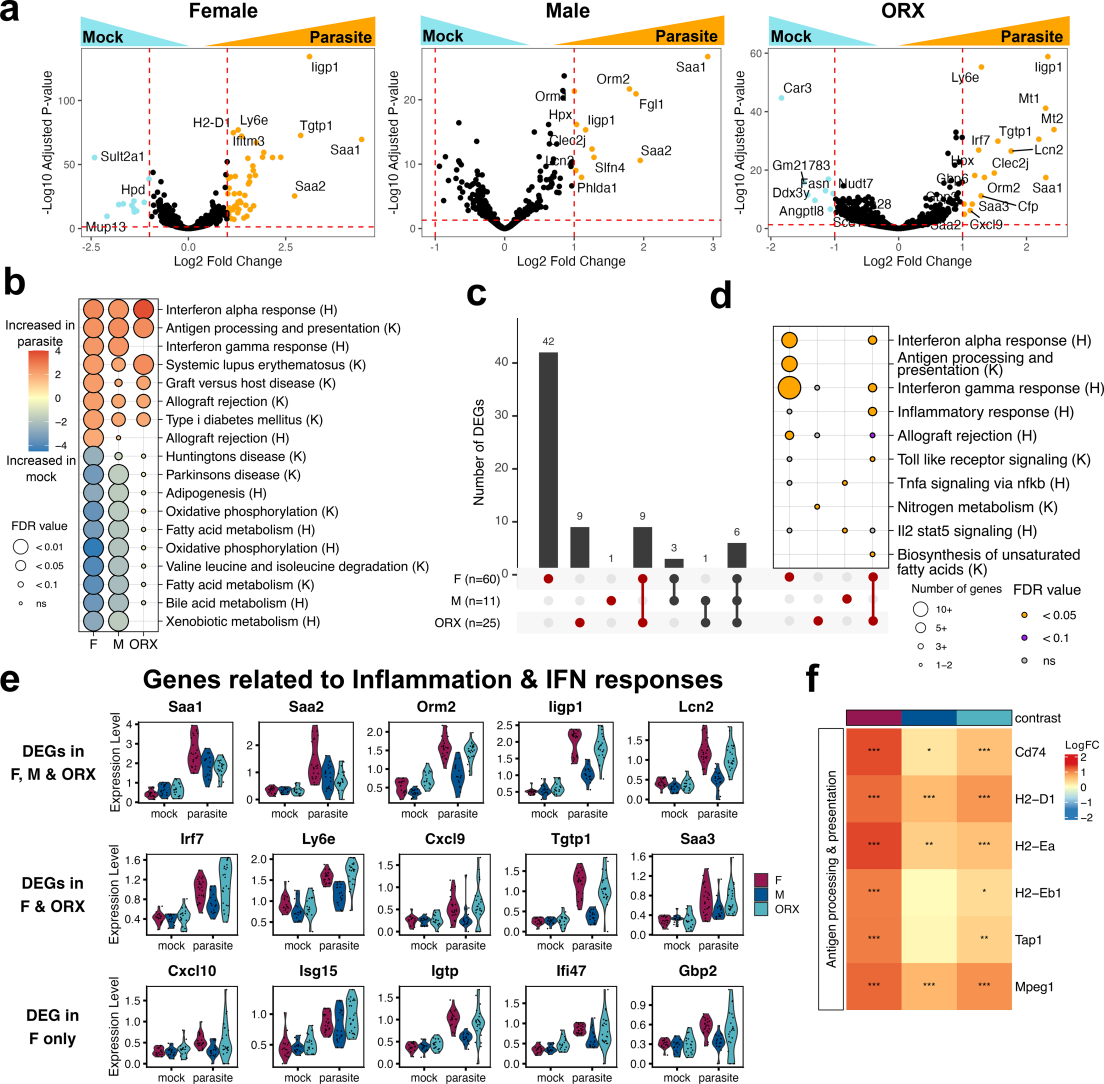
Inflammatory response to *P. yoelii* infection is influenced by sex and hormone status. **(a)** Volcano plot showing differentially expressed genes between parasite and mock-infected female (F), male (M), and orchiectomized males (ORX) mice. The x-axis shows the log_2_ fold change according to *β*_1_Equation 2. Genes significantly increased or decreased in ROIs are plotted in orange or blue, respectively (adjusted p-value< 0.05 and Log_2_ Fold Change +/- 1). **(b)** Gene set enrichment analysis (GSEA) shows pathways differentially regulated between parasite and mock for F, M, and ORX mice. H, Hallmark gene sets; K, KEGG gene sets. **(c)** Upset plot of genes shared between M/F/ORX and in response to infection (adjusted p-value < 0.05 and Log_2_ Fold Change +/- 1). **(d)** Over-representation analysis of genes unique to M, F, ORX and shared between ORX and F mice portrayed in upset plot (highlighted in red). **(e)** Violin plots of expression levels (counts per 10,000 reads) of selected genes shared between F, M, and ORX males, between F and ORX alone, and F only. **(f)** Heatmap of antigen processing and presentation related genes significantly induced in response to parasite infection. Fold change relative to mock of respective contrast (F, M, ORX) is depicted and significance is depicted by ***p.adj <0.001, **p.adj <0.01, *p.adj <0.05.

After establishing that biological sex and androgens alter innate immune cells and gene expression patterns at baseline, we aimed to evaluate whether these effects influence the host response to *Py* infection. First, we identified that *Py* infection induces a similar inflammatory state in both the ROIs around the parasite and bystander ROIs further from infected cells (**Supplementary Figure S7**). The ROIs around parasite-infected hepatocytes are also capturing uninfected cells, potentially masking a stronger differential signal to uninfected, bystander regions. To focus on the response closest to the infected-hepatocyte, we conducted pairwise comparisons between parasite and mock ROIs for female, male, and male ORX mice (**Figure 4a**; Equation 2). Differential gene expression analysis found that regardless of zonal location, IFNα responses were upregulated in infected female, males, and ORX males. Gene enrichment analysis conducted separately for females, males and ORX males confirmed enrichment of pathways previously identified as induced in response to *Pb* in female C57BL/6 mice (25). For example, all groups demonstrated an upregulation of response to type I interferons (IFNs) and antigen processing and both females and males showed downregulation of metabolism of fatty acids and bile acid in response to parasite infection (**Figure 4b**).

Further investigation in infected mice of the specific genes differentially induced between groups revealed a restricted inflammatory response within intact male mice. Overall, fewer genes were differentially induced in males, compared to female and ORX males (**Figure 4c**). While all groups demonstrated an upregulation of genes associated with cellular stress response (*Saa1, Saa2, Orm2*), the interferon response was strongest in females, intermediate in ORX males, and lowest in intact males. (**Figure 4d**). Upregulated genes at 44 hpi shared between female and ORX male mice, but not intact male mice, were linked to cellular stress responses and inflammation (e.g. *Saa3*, *Cxcl9*) and type I IFN response (e.g. *Irf7, Tgtp1, Gbp6, Ly6e*) (**Figure 4e**; **Supplementary Table S2**). At 44 hpi, females exhibited the greatest breadth of IFN response, while ORX males showed an intermediate phenotype between females and intact males (**Figure 4d**). Many differentially expressed genes in females were interferon-stimulated genes (ISGs), including transcription of *Cxcl10, Isg15*, *Bst2*, and *Ifitm3* and ISGs connected to autophagy, including *Igtp, Gbp2, Irmg1, Irmg2*, and *Ifi47* (**Figure 4e**; **Supplementary Table S2**). Thus, both female and ORX mice mount a more extensive inflammatory responses compared to intact male counterparts.

### Biological sex impacts other key pathways in response to *P. yoelii* infection

Further investigation into which immune responses are differentially regulated by biological sex revealed pathways associated with antigen processing and presentation that were more enriched within female and ORX male mice. We identified a coordinated set of expression of genes involved in antigen presentation via MHC class II (*Cd74, H2-Eb1, H2-Ea)*, MHC class I (*H2-D1, Tap1*) (**Figure 4f**), and *Mpeg1*, a macrophage-expressed gene1 that plays a central role in antigen cross-presentation in DCs (65, 66). Taken together, this may indicate either reduced frequency or function of antigen-presenting cells (APCs) and monocyte-derived APCs involved in T cell priming of CD8^+^ T cells (67, 68) in male mice.

Genes associated with iron homeostasis were also differentially induced in response to *Py* infection between sexes. *Plasmodium* parasites are known to shift iron flux during hepatocyte invasion (69). *Lsn2* was specifically increased within female and ORX male mice, which is constituently expressed primarily by neutrophils and is responsible for iron-trafficking from the cell. In contrast, *Hamp2* encoding hepcidin-2, a regulator of iron metabolism, was specifically downregulated in female mice, which could be connected to low iron levels. Interestingly, *Scd1*, a gene involved in fatty acid metabolism, was reduced within male mice, but not female and ORX male mice (**Supplementary Table S2**). Together, this data demonstrates during *Py* infection, biological sex and sex hormones influence metabolic processes related to liver stage parasite development.

While we did not identify major gene expression differences based on zonal location, we had yet to evaluate potential sex difference in zonal patterns in infection rates. We quantified the location of parasites in the liver in a separate set of validation mice that were infected with 1 x10^5^*Py* IV and harvested 44 hpi (**Supplementary Figure S8a**). Here, zonation was determined with the markers HSP70, ASS1 (periportal marker), and GS (pericentral marker) (**Supplementary Figure S8b**). Across all samples, regardless of biological sex and hormone status, there were more periportally located parasites than pericentral (**Supplementary Figure S8c**). Parasite numbers were slightly higher in the PP zone and lower in the IZ region of female mice compared to males and ORX males, with no apparent difference in the PC region. Overall, liver zonation contributes minimally to sex- and hormone-based differences, though parasites in females may be modestly skewed toward the PP zone over the IZ region.

### Biological sex impacts Kupffer cell and other immune cell responses in liver tissue

**Figure 5:**
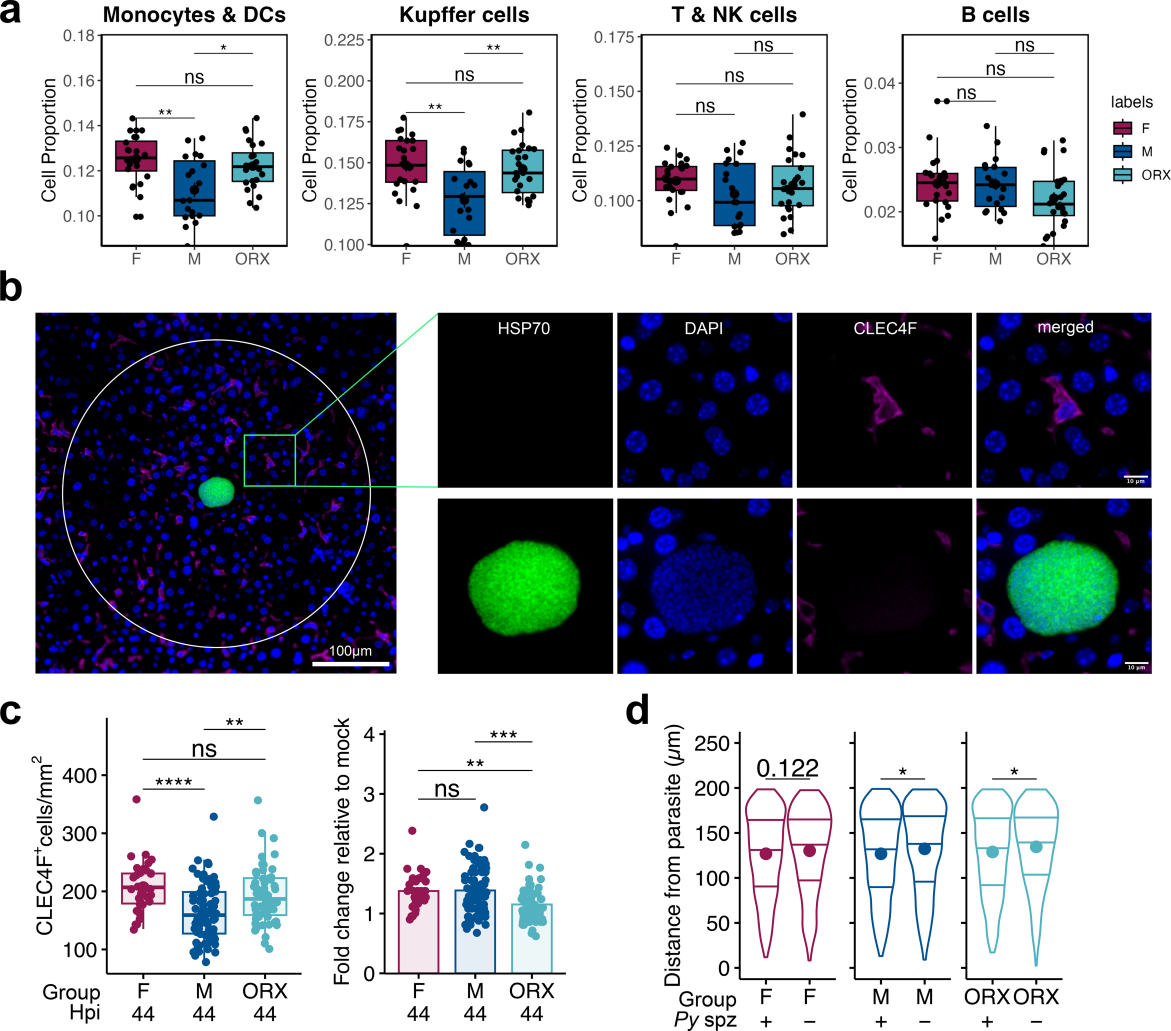
Microscopy and digital spatial profiling reveal baseline differences by sex skew enrichment of Kupffer cells and other innate immune cells. **(a)** Deconvolved immune cell type proportions for each ROI in *Py* parasite-infected mice, separated by biological sex and hormone status, inferred by spatial transcriptomic profiles. An independent Wilcoxon test with BH-correction was used to compare the mean cell type proportions between female (F), male (M), and orchiectomized male (ORX) liver samples. **(b)** Representative image of a CLEC4F^+^ positive cell proximal to parasite infected hepatocyte within an infected liver at 44 hpi. Nuclear DAPI staining is shown in blue, CLEC4F in red, and HSP70 staining in green. Ring portrays the outer boundary, indicted with white circle and captures a 200 µm radius. **(c)** Number of CLEC4F^+^ cells normalized by area of ring in infected tissue (left) and fold change of CLEC4F^+^ cells relative to respective mock-infected sample group (right) at 44 hpi. **(d)** Calculated distance of CLEC4F^+^ cell from center of ROI in infected (+) and mock-infected (-) mice. 5–10 ROI sets were counted for each of the mice. Data for c-d are shown from two independent experiments (n= 7 – 8 mice; up to 10 circles per mouse). One-way ANOVA with Tukey multiple comparison was applied for c; unpaired t-test was applied to **(d)**. Error bar represents mean ± s.e.m; box plot and violin plots depicts median with interquartile range. ****p<0.0001, ***p<0.001, **p<0.01, *p<0.05, ns p>0.05.

To identify the immune cell source driving sex-specific inflammatory response, we sought to evaluate the impact of biological sex on immune cell proportions around parasites in infected tissues. We again used the single-nucleus RNAseq data set by Hildebrandt et al. (23) to deconvolve the spatial gene expression data to estimate cell type proportion across the tissue. For this analysis, we compared immune cells between female, male and ORX male mice in the 200 µm diameter around a parasite. We found female and ORX male mice were enriched for KCs and monocytes and DCs compared to intact male mice (**Figure 5a**).

To identify if females and ORX males have higher density of KCs compared to intact males in infected tissues, we used fluorescent microscopy and quantified KCs with a CLEC4F marker in the set of mice from **Figure 1** that were infected with 1x10^5^*Py* sporozoites and harvested at 44hpi (**Figure 5b**). We further found that following infection, females and ORX males retain this increased density, while all groups increase overall density above mock-infected (**Figure 5c**). To assess differences in recruited KCs while accounting for baseline variations, we calculated the fold difference relative to mock-infected samples based on sex and hormone status. Notably, although we observed a significant decrease in KC distance from the parasite between infected and mock-infected samples, there was no significant change in the fold difference by biological sex. (**Figures 5c, d**). This data overall indicates that sex difference in KC recruitment at 44 hpi is potentially related to innate difference in baseline cell densities rather than an intrinsic activity difference between recruitment of KC toward site of infection.

## Discussion

In this study, we show that biological sex and androgens fundamentally alter the hepatic immune landscape during *Py* liver stage infection. Intact male mice exhibited higher parasite survival compared to that in females and orchiectomized (ORX) males. Using digital spatial profiling (DSP/GeoMx), we identified androgen-mediated spatial elements that impact parasite survival, including inflammatory responses, antigen presentation capacity, and baseline cellular composition. Furthermore, we defined the relative distribution of myeloid cells, with a focus on CLEC4F^+^ Kupffer cells during *Py* infection. The data suggest that these factors together influence parasite survival.

Previous studies have characterized spatial and single-cell host responses to murine *Plasmodium* spp. in female mice (23–25), establishing a baseline for hepatic immune heterogeneity. Innate immune responses in the liver are known to play a crucial role in controlling *Plasmodium* infection (26, 27, 70–72), but prior studies did not account for biological sex - a major determinant of liver metabolism and immunity in both mice (12, 18, 73) and humans (13, 74). Our findings extend this body of work by integrating sex and hormone status into the framework of liver stage immunity. Our research indicates that the male mouse liver provides a comparatively immunosuppressed microenvironment for *Py*, potentially linking hormonal modulation of immunity to parasite survival.

We propose that the diminished innate response in males reflects androgen-mediated dampening of Type I IFN signaling and immune cell recruitment, which allows *Plasmodium* parasites to better evade hepatic innate immune mechanisms in male mice. This could be due to the comparatively restricted inflammatory response linked to lower steady-state innate immune cell density in the male liver. While both sexes mount Type I IFN responses in the liver, the magnitude is markedly lower in males, consistent with the known anti-inflammatory effects of androgens in other hepatic contexts (75–77). This attenuated response may reduce hepatocyte-intrinsic clearance and myeloid activation, allowing more successful parasite development. In contrast, females and ORX males exhibited a broader, higher-density myeloid network—especially KCs and DCs, which respond to IFN signaling. In females, both hepatocyte-derived IFN production and immune cell recruitment are higher compared to males, leading to enhanced inflammatory responses. Conversely, in males, this recruitment does not occur to the same extent, resulting in improved parasite growth and development. While we did not identify the specific sources of IFNs in our study, our observations are consistent with previous research highlighting innate immune recruitment around parasites (24).

Notably, immune cell proportions were similar between parasite-containing and bystander regions on infected samples, suggesting a uniform distribution across the tissue. Our spatial data may suggest that these differences reflect baseline immune cell distributions rather than localized hotspots of inflammation, and that sex-specific hepatic immune architecture in mice sets the stage for differential parasite control. Interestingly, we observed significantly higher proportions of periportal parasites in female mice compared with both intact and orchiectomized (ORX) male mice. However, this enrichment could not be linked to an androgen-mediated outcome, as ORX males did not show a similar increase in periportal parasites. This interpretation contradicts prior studies linking the pericentral region with greater developmental success (25) and with zone-specific patterns of inflammation (23). Several factors may account for this discrepancy, including differences in *Plasmodium* strain, mouse strain, and the limited spatial resolution of the GeoMX DSP platform, which does not achieve single-cell resolution.

Beyond inflammation, our data point to three androgen-sensitive pathways that may enhance parasite clearance in an androgen-low environment (1): increased hepatocyte-intrinsic abortive clearance mechanisms (2), increased antigen processing and presentation, and (3) reduced metabolic sequestration and nutrient uptake by the parasite, thus limiting development and leading to clearance. First, abortive hepatocytes, cells that autonomously clear infection (78–80), may be more functional in female mice and more limited in androgen-high males. We found higher expression of abortive-like gene expression (*Cxcl10*, *Tgtp1*) (25) in infected tissue from females and ORX males versus intact males, suggesting enhanced cell-autonomous clearance capacity.

Second, transcriptional upregulation of antigen-processing and presentation genes in females and ORX males indicates more effective adaptive immune potential—consistent with evidence that reduced androgen receptor signaling enhances MHC I expression (81) and with reports of sex-specific vaccine efficacy (36). Future studies could formally test whether the intrinsic evasion mechanisms employed by the parasite are better suited to a testosterone-rich host liver where anti-inflammatory mechanisms limit activation of genes contributing to cell-autonomous clearance mechanisms.

Third, previous studies have speculated iron handling and lipid/fatty acid metabolism in facilitating *Plasmodium* liver stage development. In our data, we observed larger hepatocyte size in males versus females at 24 hpi, but not 44 hpi, implicating a potential sex-specific effect on early replication. When measured at 44 hpi, genes connected to iron flux in hepatocytes, *Hamp2* and *Lcn2* (82, 83), have reduced expression in intact male mice compared to females and ORX males. Iron overload has been shown to favor *Py* intrahepatic development (84), so future work should investigate a potential connection between parasite development and sex-specific iron homeostasis. We observed baseline differences in lipid and fatty acid metabolism gene expression (i.e., Cyp2b13), consistent with known sex-specific pathways. While prior studies show that elevated lipid metabolism in female mice creates an anti-inflammatory environment around schizonts (23), we did not detect this in our dataset. Interestingly, we detected upregulation of genes involved in the endocrine system in female mice only. One set of genes were *G6pc*, a glucose-6-phosphatase, and *Sult2a1*, a sulfotransferase known to metabolize dehydroepiandrosterone (DHEA), an androgen, to DHEA-S in the liver (85). The liver is the main site of conversion of DHEA to its’ secretory form DHEA-S, and this conversion seems to be down regulated in response to *Plasmodium* infection (86). Together, these findings suggest that female and ORX mice sustain a more metabolically active environment during infection that may link with effective parasite clearance and anti-evasion responses. However, direct causal links and temporal dynamics of these genes remain to be established and warrant further investigation.

Ultimately our study adds another dimension to the current benchmark for host-pathogen interactions in the *Plasmodium*-infected mouse liver. Cell type deconvolution found higher proportion of monocytes, DCs, and Kupffer cells in infected samples compared to mock-infected samples. This is in contrast to Hildebrandt et al. (23), which found no significant shift in cell proportions in infected samples compared to a salivary-gland lysate injection control. We used mock-infected controls by design to capture baseline differences between groups; however, because the salivary gland lysate may itself induce inflammation, we cannot exclude nonspecific effects. Another limitation is that the reported proportions reflect transcripts rather than cell densities. Use of a snRNA-seq reference on probe-targeted GeoMx DSP data introduces modality and platform biases (nuclear vs. cytoplasmic representation, probe coverage, capture efficiency), and ROI composition (cell size, shape, hepatocyte polyploidy) can further skew absolute fractions. We therefore prioritize microscopy-validated measurements and interpret deconvolution cautiously. Future studies should examine earlier timepoints, incorporate higher spatial resolution, and incorporate parasite transcriptomics to uncover sex-specific host-parasite interactions shaping innate immune responses.

This study integrates biological sex into the spatial framework of *Plasmodium* liver stage infection, revealing that androgen signaling modulates both the architecture and transcriptional tone of hepatic immunity. By linking sex hormones to spatial immune restraint, this work adds a key dimension to liver stage malaria biology. Understanding how hormonal environments reshape parasite-host equilibrium will be critical for designing vaccines and therapeutics that achieve consistent protection across diverse human populations. Future research should translate these findings to human systems, where comparable sex differences in hepatic metabolism and immunity may influence infection outcomes and vaccine efficacy.

## Data Availability

The GeoMX DSP raw and processed data reported in this paper is deposited in Zenodo (10.5281/zenodo.18235390). Additional data files and analysis scripts are available at [https://github.com/carduncombe/Duncombe-et-al-Front-Immunol-2026.git].
