# Supplementary material for: Host biological sex directs immune control of the Plasmodium parasite liver stage in mice
Source: Front Immunol. 2026 Mar 2;17:1734587. doi: 10.3389/fimmu.2026.1734587 (PMC12989532; doi:10.3389/fimmu.2026.1734587)
Supplement: Supplementary file 1 [file DataSheet1.pdf]

Supplemental Figures

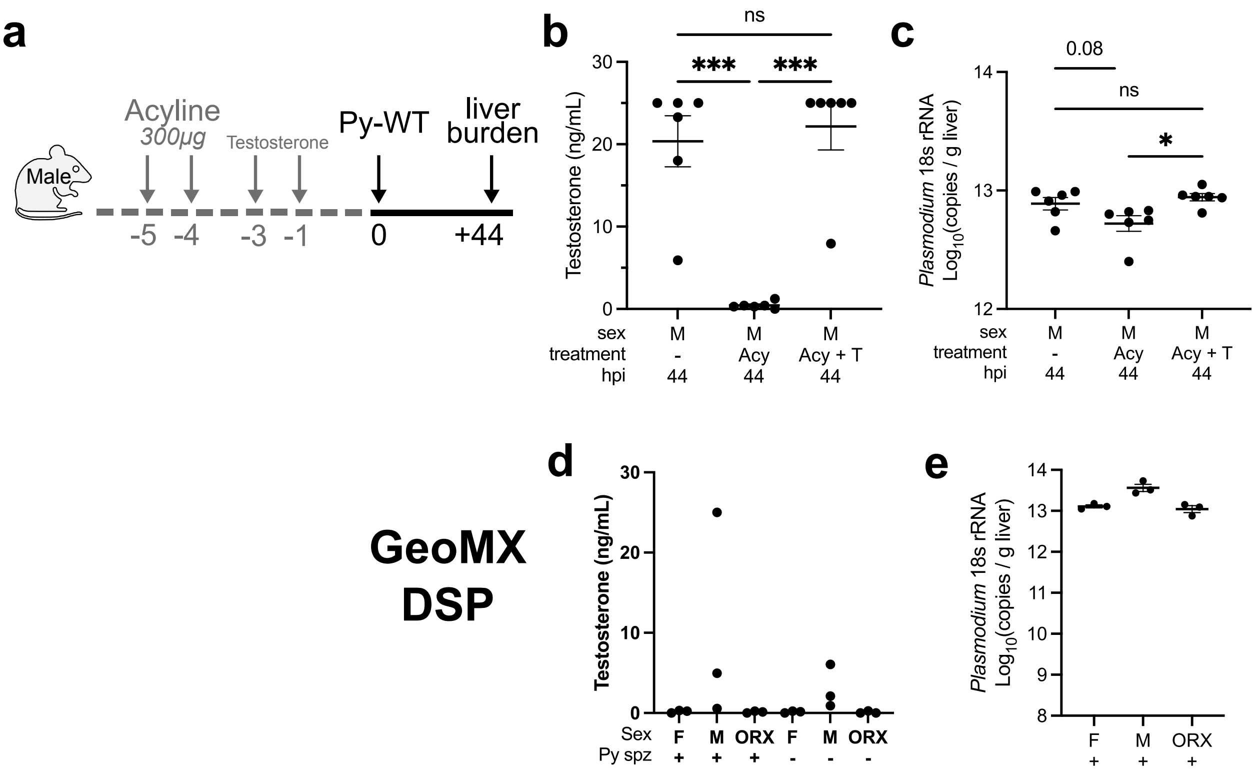

Supplemental Figure 1: Testosterone alters liver burden to *Plasmodium yoelii* infection in BALB/cJ mice.
a. Scheme of male (M) BALB/cJ mice infected with $1x10^5$ *Py*-WT spz intravenously (IV) and harvested at 44 hours post infection (hpi). M mice received two doses of 300 µg acyline (Acy), two doses of Acy (300µg) plus two doses of testosterone (T - 100µg/g), or left untreated. **b.** Confirmatory testosterone measurements by ELISA of the mice. **c.** Liver burden at 44 hpi as absolute pan-*Plasmodium* 18S rRNA copy numbers. Data are shown from two independent experiments (n= 6/group). Statistical significance was determined by one-way ANOVA with Tukey multiple comparison. ***p <0.001 and ns p>0.05. **d.** Confirmatory testosterone measurements by ELISA of the mice in GeoMX experiment in **Figures 2–5**. **e.** Liver burden of GeoMX experiment at time of collection (44hpi) as absolute pan-*Plasmodium* 18S rRNA copy numbers. Data depict one independent replicate (n=3/group). Error bar represents mean ± s.e.m.

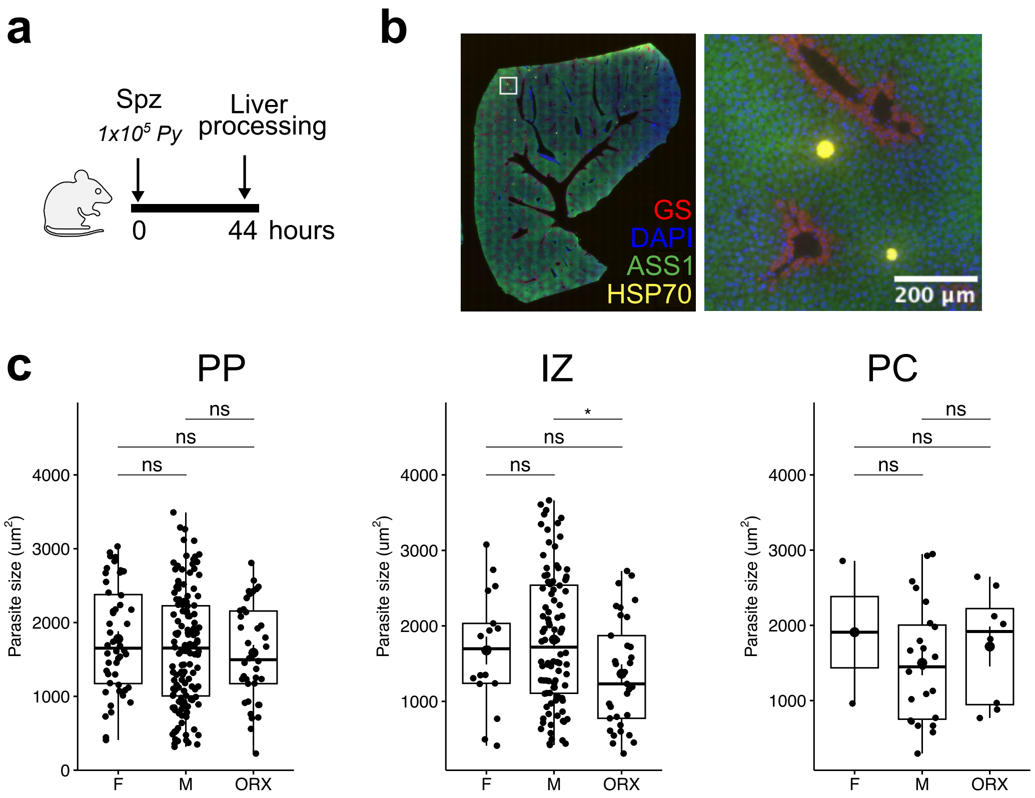

Supplemental Figure 2: Size of parasite-infected hepatocytes by zonal location. a. Scheme of male (M), female (F), and male orchiectomized (ORX) BALB/cJ mice infected with $1x10^5$ *Py*-WT spz intravenously (IV) and harvested at 44 hours post infection (hpi). **b.** Representative image of the size variability of the parasite in the liver. Periportal (PP), interzonal (IZ), and pericentral (PC) were determined with the markers GS (red), ASS1(green), and DAPI (blue) and size was determined with *Plasmodium* marker HSP70 (yellow). **c.** Size of parasite-infected hepatocytes within the PP (left), IZ(middle), and PC (right) region of the liver as determined by fluorescent microscopy. Statistical significance was determined by one-way ANOVA with Tukey multiple comparison. Data are shown from two independent experiments (n= 7-8/group). Boxplots depict median with interquartile range. *p<0.05 and ns p>0.05.

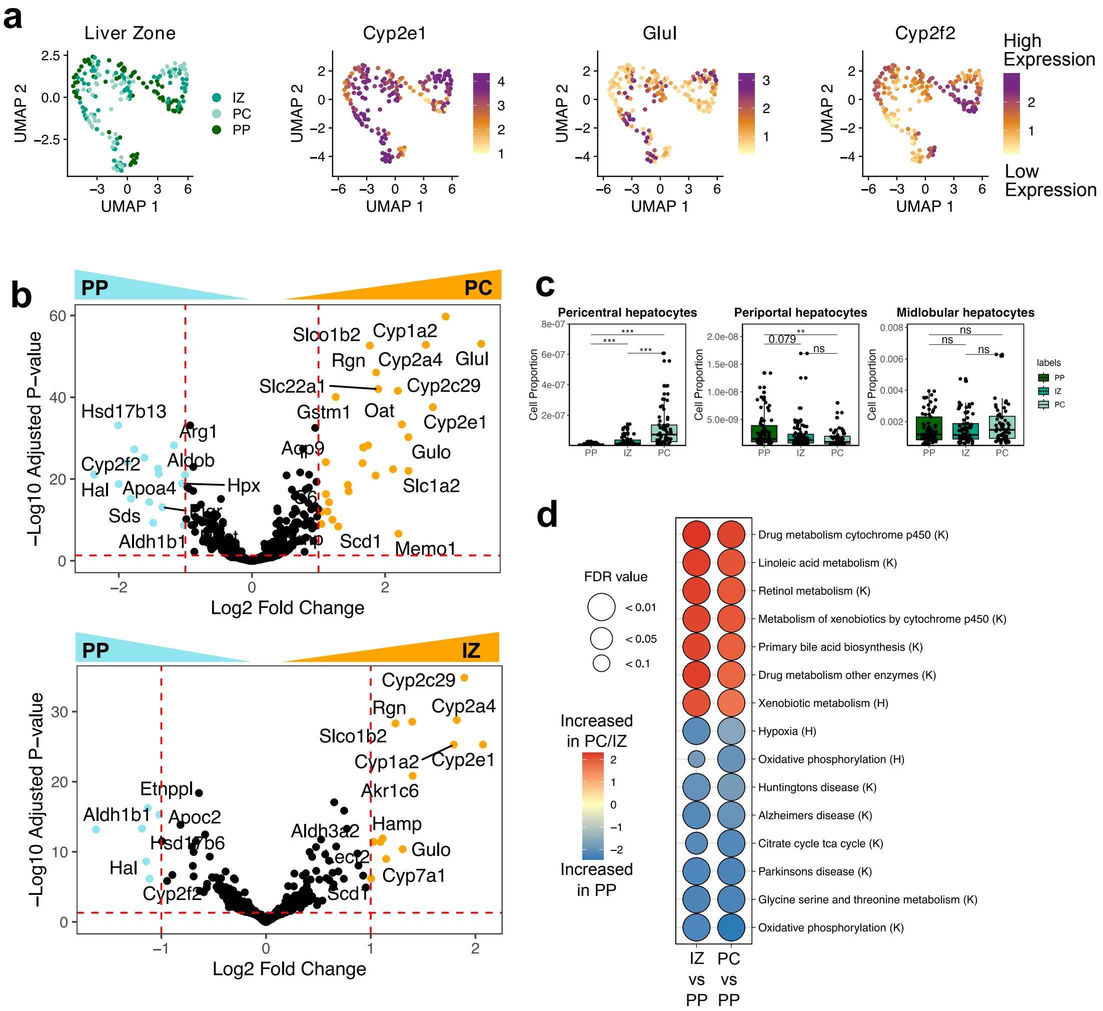

Supplemental Figure 3: Gene expression confirms accurate differentiation of liver zones during regions of interest (ROIs) selection. a. UMAP of ROIs, colored by liver zone (left), and by expression of the pericentral (PC) genes *Cyp2e1* and *Glul*, and periportal (PP) gene *Cyp2f2* (right). **b.** Volcano plot of differentially expressed genes conserved between PP region or interzonal (IZ) region and PC region. The y-axis shows the $\log_{10}$ of the adjusted p-value (BH-corrected). The x-axis shows the $\log_2$ fold change according to β_3, Equation 1. Genes significantly increased or decreased in ROIs are plotted in orange or blue, respectively (adjusted p-value< 0.05 and Log_2 Fold Change +/- 1). Gene symbols are shown for selected genes. **c.** Calculated immune cell type proportions for each ROI in mock-infected mice, separated by liver zone. An independent Wilcoxon test was used to compare the mean cell type proportions between female, male, and ORX male samples, and the resulting p-values are shown. Boxplots depict median with interquartile range. ***p <0.001, **p<0.01, *p<0.05, ns p>0.05. **d.** Gene set enrichment analysis (GSEA) shows pathways differentially regulated between IZ and PP, and PC and PP. H, Hallmark gene sets; K, KEGG gene sets. Color scale denotes the Normalized Enrichment Score (NES).

a

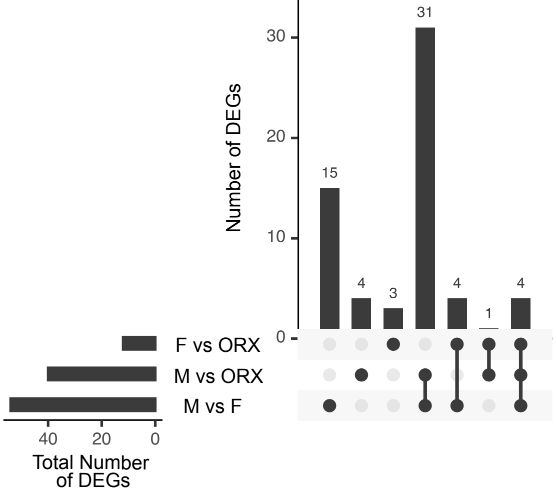

b

Comparison	Number of genes	DEGs
M vs F	15	Actg1, Aldh1a1, C6, C8a, Cbr1, Comt, Cyp2a4, Cyp2d12, Cyp2f2, Cyp8b1, Keg1, Lrg1, Mfsd2a, Sult1a1, Sult2a8
M vs ORX	4	Bhmt, H2-Ea, Orm2, Phyh
F vs ORX	3	Ddx3y, Gm21783, Igfbp2
Shared M vs F & M vs ORX	31	Acot3, Aox3, C4a, C8b, C9, Car3, Csad, Cyp2b13, Cyp2c37, Cyp2d10, Cyp4a10, Cyp4a12a, Cyp4a14, Cyp7b1, Egfr, Fgl1, Fmo3, Gstp1, Hamp2, Hao2, Hsd3b5, Nudt7, Prlr, Scp2, Selenbp1, Serpina1a, Serpina3k, Slc22a27, Slco1a1, Ttc39c, Ugt2b1
Shared M vs F & F vs ORX	4	Cyp7a1, Mt1, Mt2, Mup13
Shared M vs ORX & F vs ORX	1	Scd1
Shared between all	4	Ces3b, Sult2a1, Sult2a2, Sult2a5

Supplemental Figure 4: Differentially expressed genes between steady state female (F), male (M), and orchiectomized male (ORX) mice. a. Upset plot of differentially expressed genes shared between groups (adjusted p-value < 0.05 and Log_2 Fold Change +/- 1) from comparisons plotted in **Figure 3c**. **b.** Table of differentially expressed genes for each comparison in **Figure 3c.**

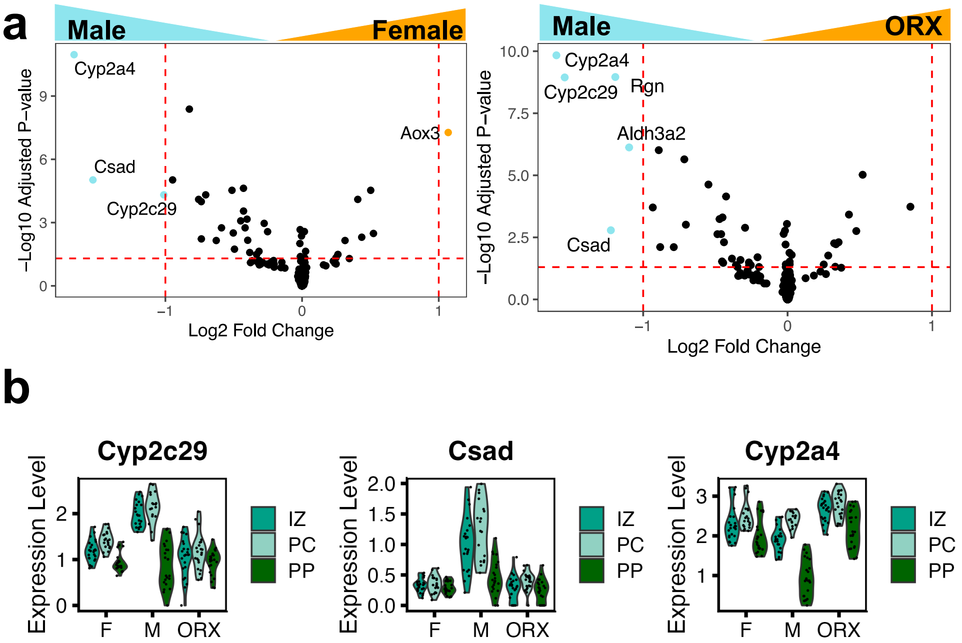

Supplemental Figure 5: Biological sex and hormone status minimally perturb gene expression profiles of liver zones at GeoMX resolution. a. Volcano plot of showing differentially boosted genes conserved between male (M) and females (F) (left) or M and orchiectomized males (ORX) (right) in the context of genes differentially expressed between periportal (PP) and pericentral (PC). The y-axis shows the $\log_{10}$ of the adjusted p-value (BH-corrected). The x-axis shows the $\log_2$ fold change according to β_5, Equation 1. Genes significantly increased or decreased in ROIs are plotted in orange or blue, respectively (adjusted p-value< 0.05 and Log_2 Fold Change +/- 1). Gene symbols are shown for significant genes. **b.** Violin plots of normalized and batch-corrected gene expression of gene *Cyp2c29*, *Csad*, and *Cyp2a4*.

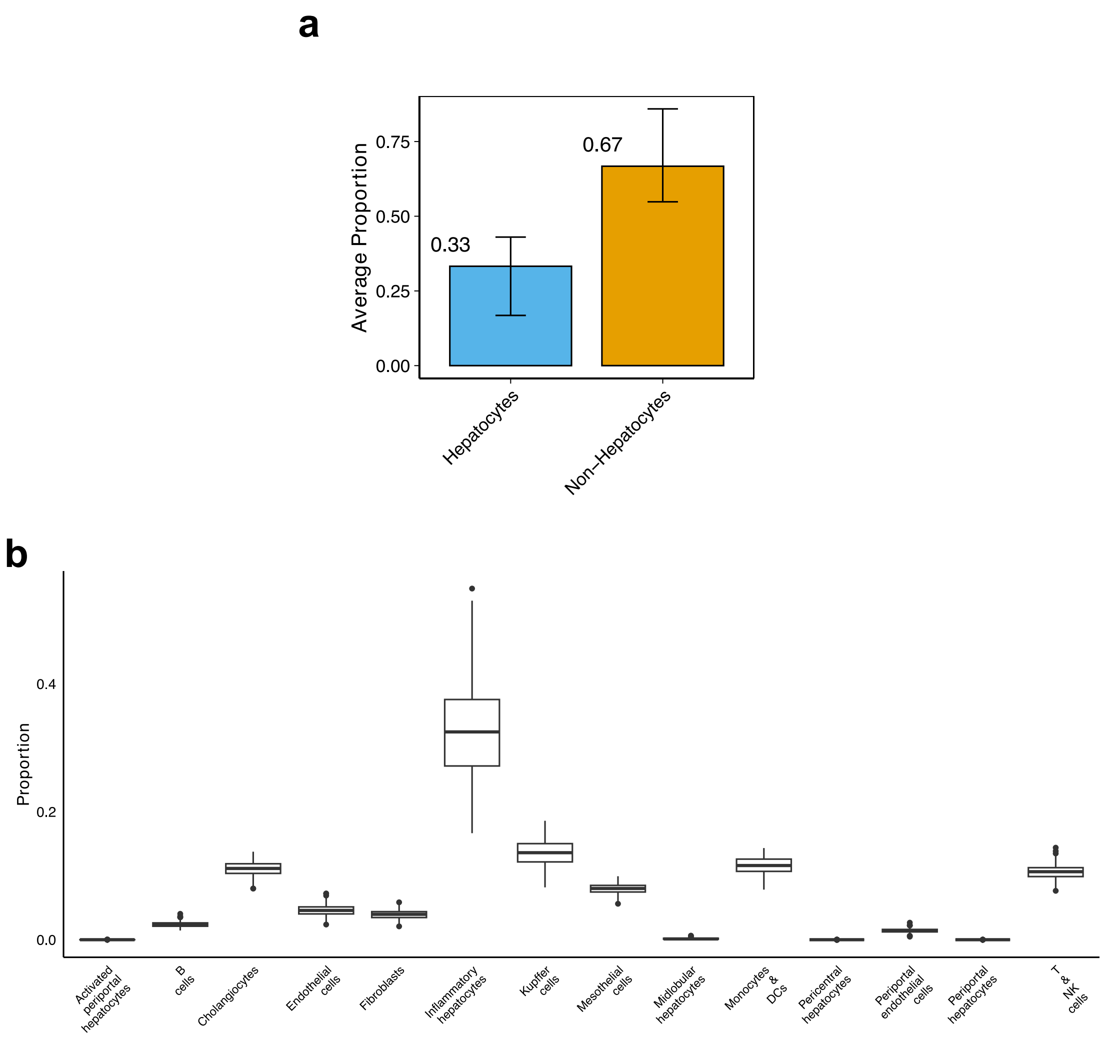

Supplemental Figure 6: Proportions of cell types across all samples. **a**. Average proportion of hepatocytes across all conditions compared to the average proportion of all other cell types identified by cellular deconvolution (error bars correspond to the upper and lower range). **b**. Boxplots of cell proportions of all cell types across all conditions in **Figure 2a**. Boxplots depict median with interquartile range (n = 18 mice).

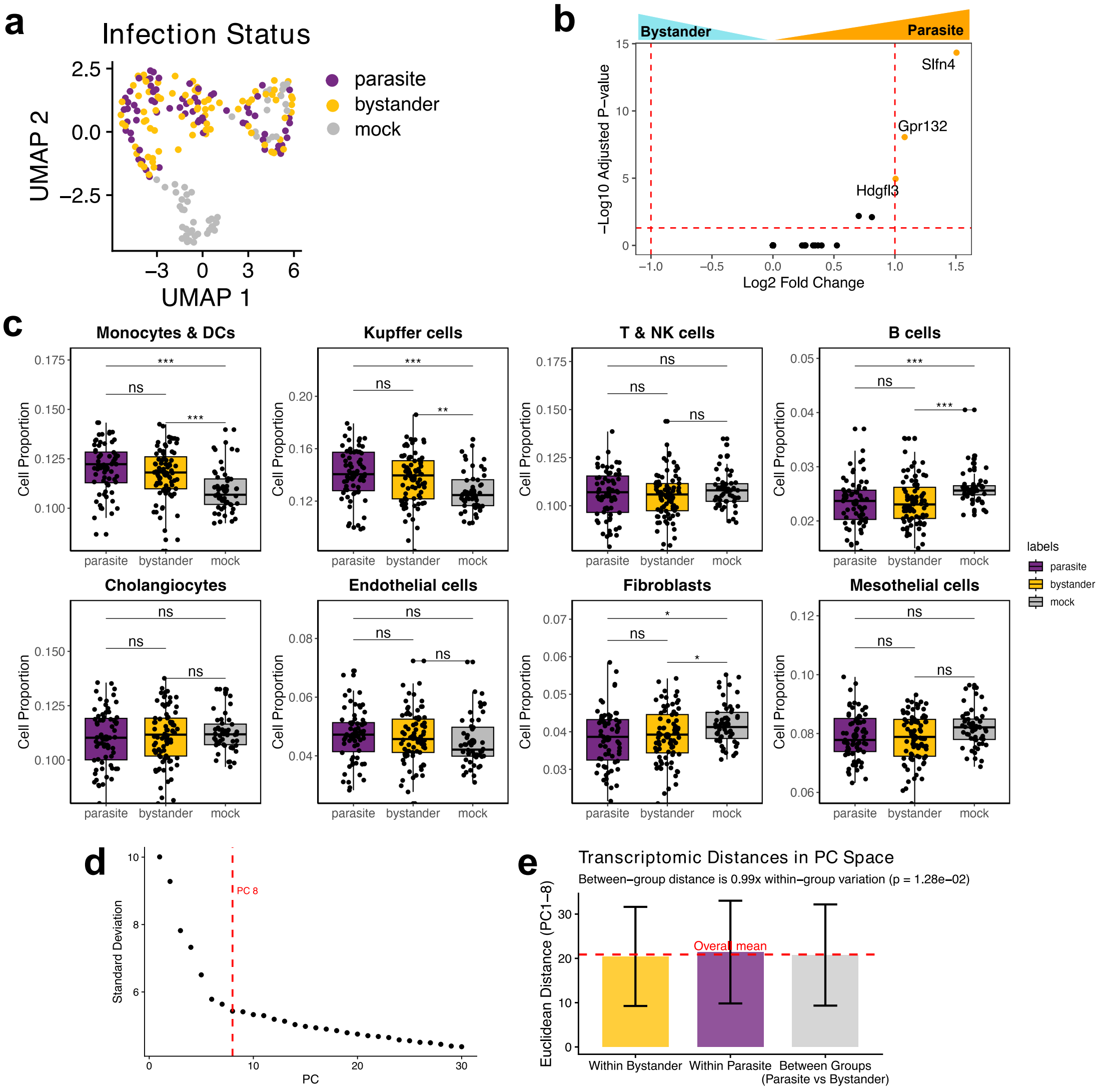

Supplemental Figure 7: *P. yoelii* infection impacts proximal and peripheral immune cell recruitment and genes expression in liver tissue. a. UMAP of ROIs, colored by infection type (parasite, bystander, or mock). **b.** Volcano plot of differentially boosted genes between parasite and bystander to parasite. The y-axis shows the $\log_{10}$ of the adjusted p-value (BH-corrected). The x-axis shows the $\log_2$ fold change according to β_2, Equation 1. Gene significantly increased or decreased in ROIs are plotted in orange or blue, respectively (adjusted p-value< 0.05 and Log_2 Fold Change +/- 1). Gene symbols are shown for selected genes. **c.** Calculated immune cell type proportions for each ROI in all mice separated by infection type. An independent Wilcoxon test (BH-corrected) compared the mean cell type proportions and the resulting p-values are shown. ***p <0.001, **p<0.01, *p<0.05, ns p>0.05. **d.** Elbow plot depicts the standard deviation of all included ROIs explained by principal components. Eight PCs were retained for dimensionality reduction, capturing the majority of variance while minimizing noise. **e.** Euclidean distances in principle-component space (PC1–8) between parasite proximal and bystander ROIs compared with within-group distances. Between bystander and parasite = 20.8 ± 11.4; within bystander = 20.4 ± 11.1; within parasite = 21.4 ± 11.6 (Kruskal–Wallis p = 1.28e-02). Error bar represents mean ± s.e.m.

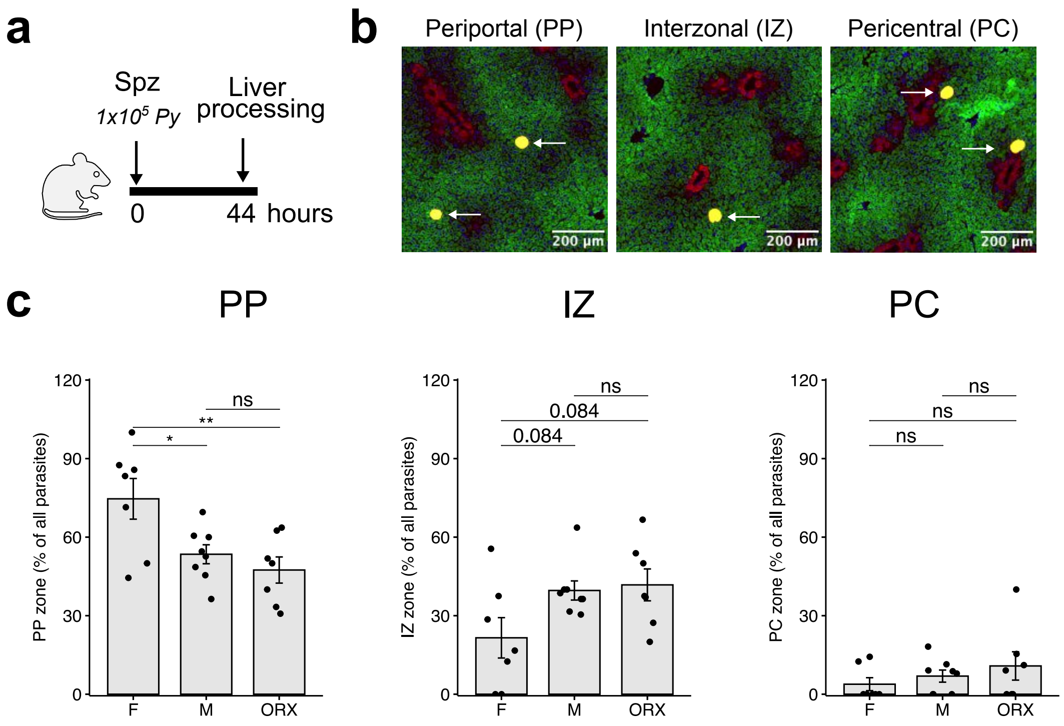

Supplemental Figure 8: Impact of sex and orchiectomy on zonation of *Plasmodium yoelii* infection in the liver. a. Scheme of female (F), male (M) and male orchiectomized (ORX) BALB/cJ mice infected with $1x10^5$ *Py*-WT spz intravenously (IV) and harvested at 44 hours post infection (hpi). **b.** Representative image of the location of the parasite in the liver. Periportal (PP), interzonal (IZ), and pericentral (PC) were determined with the markers GS (red), ASS1(green), HSP70 (yellow), and DAPI (blue). **c.** Percentage of total parasite-infected hepatocytes within the PP (left), IZ (middle), and PC (right) region of the liver as determined by fluorescent microscopy. Statistical significance for data was determined by one-way ANOVA with Tukey multiple comparison. Data are shown from two independent experiments (n= 7-8/group). Error bar represents mean ± s.e.m. $**p<0.01$, $*p<0.05$, ns $p>0.05$.

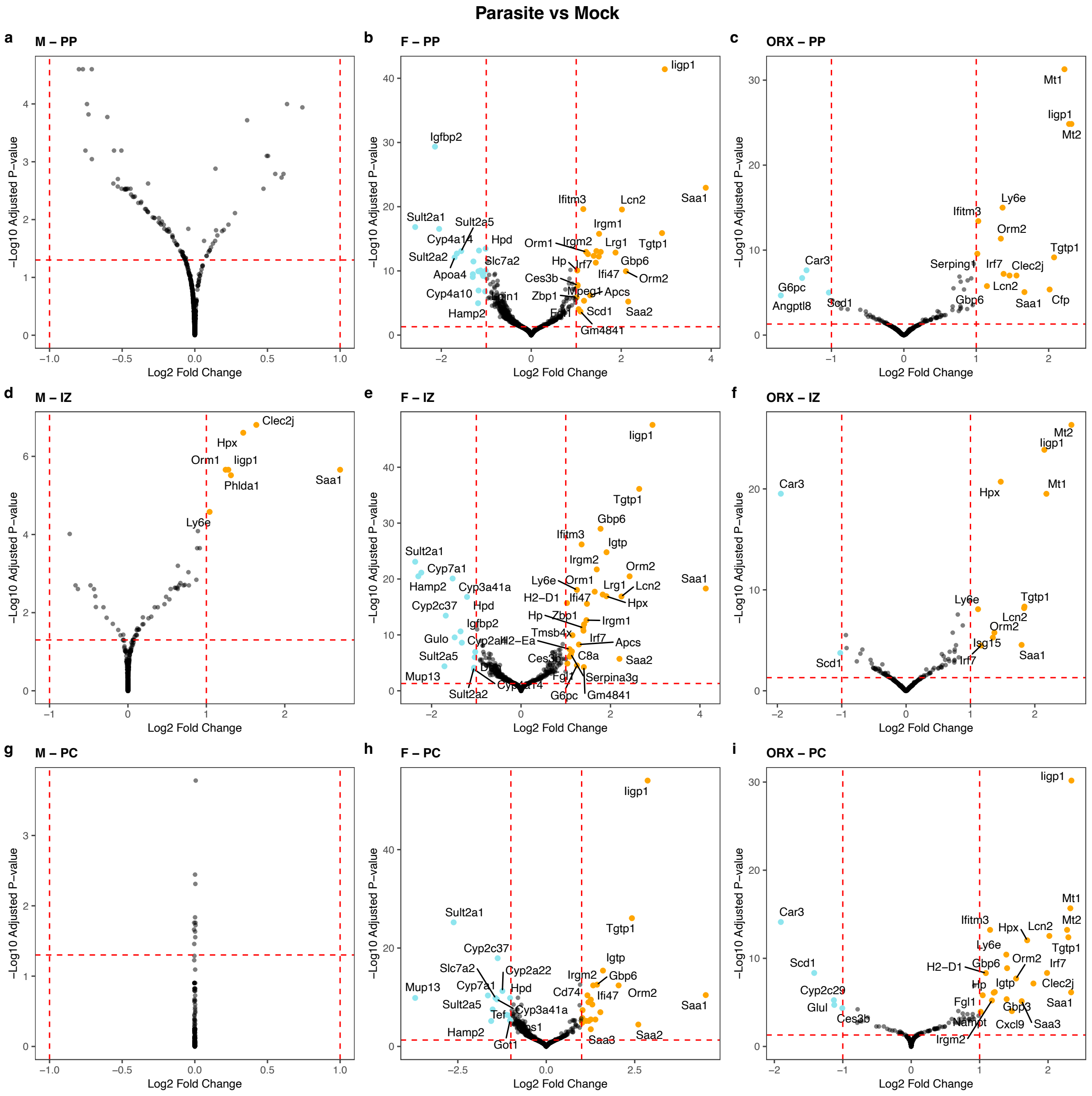

Supplemental Figure 9: Parasite vs Mock Volcano Plot. a – i. Volcano plot of differentially boosted genes between parasite and mock ROIs. The y-axis shows the $\log_{10}$ of the adjusted p-value (BH-corrected). The x-axis shows the $\log_2$ fold change. Gene significantly increased in parasite or decreased in parasite ROIs are plotted in orange or blue, respectively (adjusted p-value< 0.05 and Log_2 Fold Change +/- 1). Gene symbols are shown for selected genes.

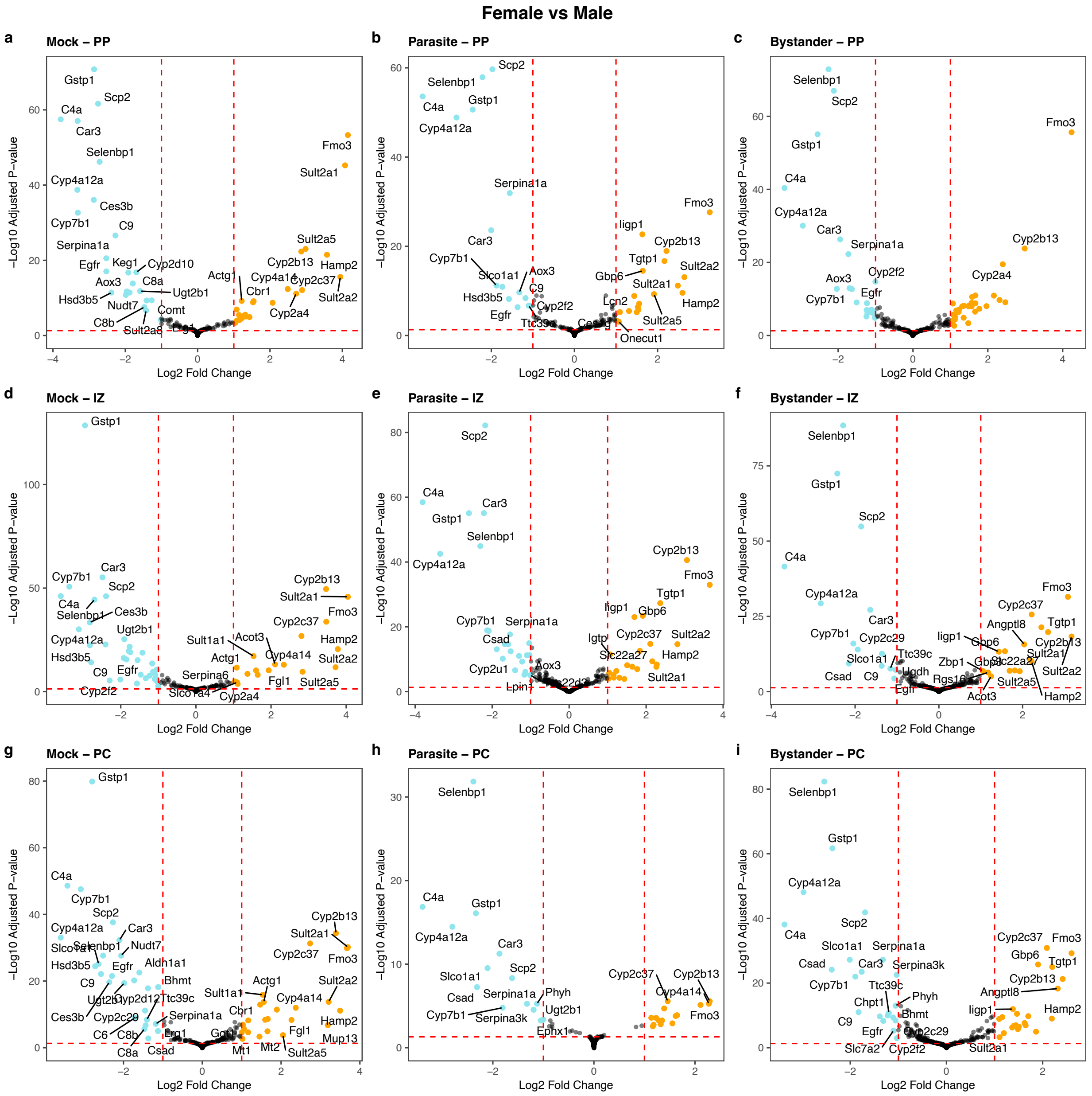

Supplemental Figure 10: Female vs Male Volcano Plots. a – i. Volcano plot of differentially boosted genes between female and male mice. The y-axis shows the $\log_{10}$ of the adjusted p-value (BH-corrected). The x-axis shows the $\log_2$ fold change. Gene significantly increased in females or increased in male mice are plotted in orange or blue, respectively (adjusted p-value< 0.05 and Log_2 Fold Change +/- 1). Gene symbols are shown for selected genes.

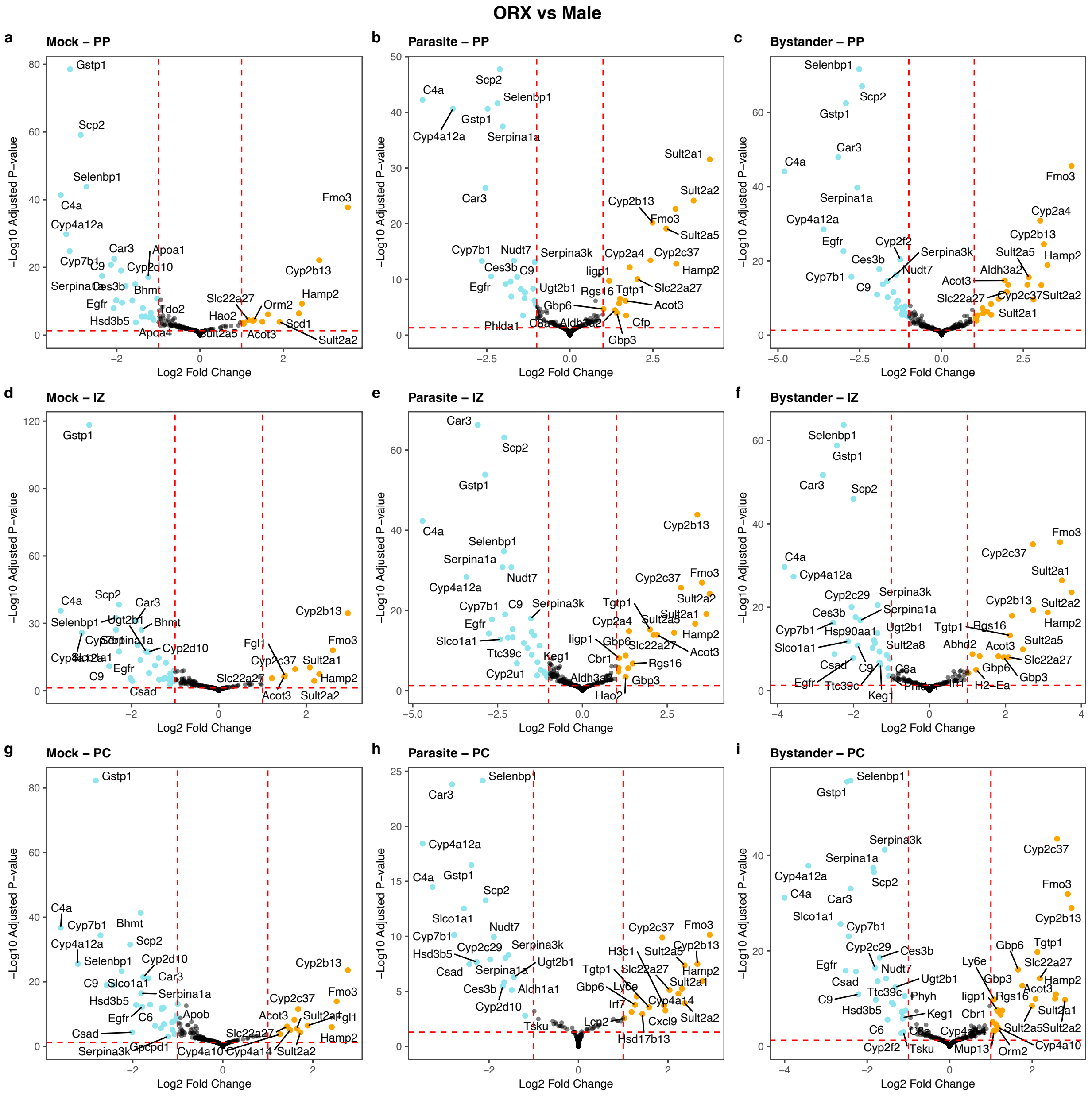

Supplemental Figure 11: ORX Male vs Intact Male Volcano Plots. a – i. Volcano plot of differentially boosted genes between ORX male and intact male mice. The y-axis shows the $\log_{10}$ of the adjusted p-value (BH-corrected). The x-axis shows the $\log_2$ fold change. Gene significantly increased in ORX males or increased in intact male mice are plotted in orange or blue, respectively (adjusted p-value< 0.05 and Log_2 Fold Change +/- 1). Gene symbols are shown for selected genes.

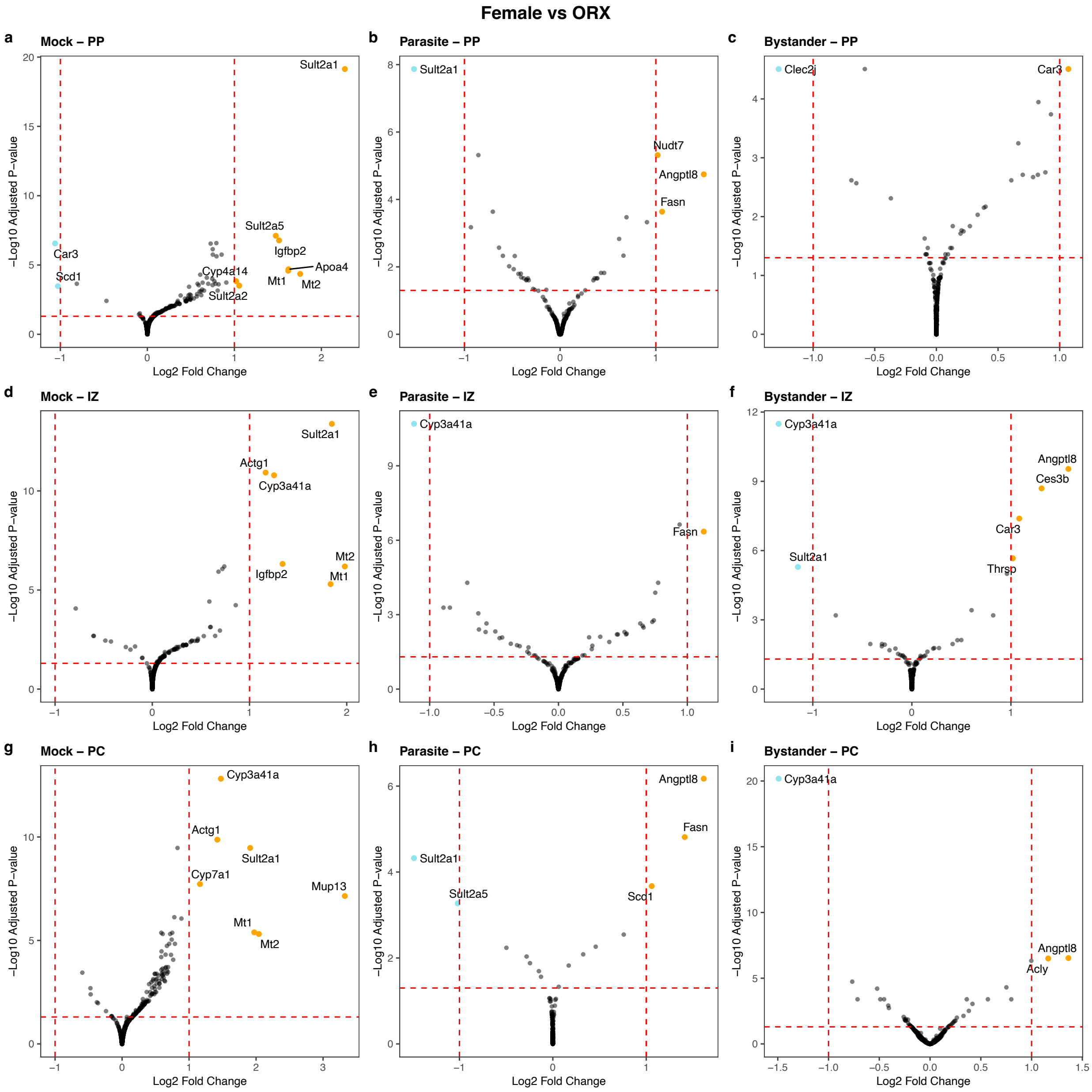

Supplemental Figure 12: Female vs ORX Male Volcano Plots. a – i. Volcano plot of differentially boosted genes between female and ORX male mice. The y-axis shows the $\log_{10}$ of the adjusted p-value (BH-corrected). The x-axis shows the $\log_2$ fold change. Gene significantly increased in female or increased in ORX male mice are plotted in orange or blue, respectively (adjusted p-value< 0.05 and Log_2 Fold Change +/- 1). Gene symbols are shown for selected genes.

Supplemental Table 1: Summary table of final number of regions of interest (ROIs) per condition following quality control and exclusion criteria. “Parasite” refers to an ROI centered on a parasite-infected hepatocyte in an infected mouse. “Bystander” refers to an ROI in an infected mouse that does not encompass a parasite-infected hepatocyte. “Mock” refers to an ROI in an uninfected, mock-injected mouse. Biological sex and hormone status variables: F = female, M = male, ORX = orchiectomized male. Liver zone variables: PP = periportal, IZ = interzonal, PC = pericentral.

		PP	IZ	PC
Parasite	**F**	9	9	6
	**M**	9	7	3
	**ORX**	8	9	7
Bystander	**F**	8	9	9
	**M**	9	9	9
	**ORX**	9	9	9
Mock	**F**	6	6	5
	**M**	5	6	6
	**ORX**	6	6	6
			**Total**	**199**

Supplemental Table 2: Differentially expressed genes (DEGs) between mock and parasite regions of interest (ROI) for β_1, Equation 2.

Comparison (Parasite vs. mock)	Number of genes	DEGs
ORX	9	Angptl8, Car3, Cfp, Ddx3y, Fasn, Mt1, Mt2, Nudt7, Slc22a28
F	42	Apcs, Apol9b, Bst2, Cd74, Ces3b, Cxcl10, Cyp2c37, Cyp3a41a, Cyp4a14, Cyp7a1, G6pc, Gbp2, Gbp2b, Gm20792, Gm4841, H2-D1, H2-Ea, H2-Eb1, H2-K1, Hamp2, Hp, Hpd, Ifi47, Ifitm3, Igfbp2, Igtp, Irgm1, Irgm2, Isg15, Lrg1, Mpeg1, Mup13, Psmb8, Serpina10, Serpina3g, Steap4, Sult2a1, Sult2a2, Sult2a5, Tap1, Tmsb4x, Zbp1
M	1	Phlda1
Shared between F & ORX	9	Cxcl9, Gbp3, Gbp6, Gm21783, Irf7, Ly6e, Saa3, Scd1, Tgtp1
Shared between M & ORX	1	Clec2j
Shared between F & M	3	Fgl1, Orm1, Slfn4
Shared between all	6	Hpx, Iigp1, Lcn2, Orm2, Saa1, Saa2
